# Human cytomegalovirus exploits STING signaling and counteracts IFN/ISG induction to facilitate infection of dendritic cells

**DOI:** 10.1038/s41467-024-45614-3

**Published:** 2024-02-26

**Authors:** Bibiana Costa, Jennifer Becker, Tobias Krammer, Felix Mulenge, Verónica Durán, Andreas Pavlou, Olivia Luise Gern, Xiaojing Chu, Yang Li, Luka Čičin-Šain, Britta Eiz-Vesper, Martin Messerle, Lars Dölken, Antoine-Emmanuel Saliba, Florian Erhard, Ulrich Kalinke

**Affiliations:** 1 Institute for Experimental Infection Research, TWINCORE, Centre for Experimental and Clinical Infection Research, a joint venture between the Helmholtz Centre for Infection Research and the Hannover Medical School, Hannover, Germany; 2 Helmholtz Institute for RNA-based Infection Research (HIRI), Helmholtz-Centre for Infection Research (HZI), Würzburg, Germany; 3 Department of Computational Biology for Individualised Medicine, Centre for Individualised Infection Medicine (CiiM) & TWINCORE, a joint venture between the Helmholtz Centre for Infection Research and the Hannover Medical School, Hannover, Germany; 4 Department of Internal Medicine and Radboud Institute for Molecular Life Sciences, Radboud University Medical Center, Nijmegen, the Netherlands; 5 Institute for Immune Aging and Chronic Infection, Helmholtz Centre for Infection Research, Braunschweig, Germany; 6 Institute for Transfusion Medicine and Transplant Engineering, Hannover Medical School, Hannover, Germany; 7 Institute of Virology, Hannover Medical School, Hannover, Germany; 8 Institute for Virology and Immunobiology, University of Würzburg, Würzburg, Germany; 9 University of Würzburg, Faculty of Medicine, Institute of Molecular Infection Biology (IMIB), Würzburg, Germany; 10 Faculty for Informatics and Data Science, University of Regensburg, Regensburg, Germany; 11 Cluster of Excellence - Resolving Infection Susceptibility (RESIST, EXC 2155), Hannover Medical School, Hannover, Germany

**Keywords:** Interferons, Herpes virus, Virus-host interactions, Dendritic cells

## Abstract

Human cytomegalovirus (HCMV) is a widespread pathogen that in immunocompromised hosts can cause life-threatening disease. Studying HCMV-exposed monocyte-derived dendritic cells by single-cell RNA sequencing, we observe that most cells are entered by the virus, whereas less than 30% of them initiate viral gene expression. Increased viral gene expression is associated with activation of the stimulator of interferon genes (STING) that usually induces anti-viral interferon responses, and with the induction of several pro- (*RHOB, HSP1A1, DNAJB1*) and anti-viral (*RNF213, TNFSF10, IFI16*) genes. Upon progression of infection, interferon-beta but not interferon-lambda transcription is inhibited. Similarly, interferon-stimulated gene expression is initially induced and then shut off, thus further promoting productive infection. Monocyte-derived dendritic cells are composed of 3 subsets, with one being especially susceptible to HCMV. In conclusion, HCMV permissiveness of monocyte-derived dendritic cells depends on complex interactions between virus sensing, regulation of the interferon response, and viral gene expression.

## Abstract (web summary)

Human cytomegalovirus (HCMV) is a ubiquitous pathogen associated with morbidity and mortality in the immunocompromised or immunonaive context. Here the authors show that HCMV exploits STING signalling and subverts the interferon response to support infection of monocyte derive dendritic cells.

## Introduction

Human cytomegalovirus (HCMV) is a human-specific β-herpesvirus with a prevalence of 40-90% in the human population. While in immunocompetent hosts HCMV infection is mostly sub-clinical, immunocompromised individuals may develop life-threatening disease. Furthermore, HCMV is the leading cause of congenital disabilities^1^. Myeloid cells are natural targets of lytic and latent HCMV infection in vivo and represent an important viral reservoir^2,3^. Monocytes are recruited from the blood to sites of inflammation, where they differentiate to macrophages and/or dendritic cells (DCs)^4,5^. DCs prime and re-stimulate HCMV-specific T cells and are important producers of interferons (IFN), which induce the expression of anti-viral IFN-stimulated genes (ISGs) that protect the host from severe HCMV infection^6^. HCMV evades and exploits DC functions^7^, re-purposes anti-viral ISGs to enhance its own replication (reviewed in^8^) and encodes several proteins that are dedicated to the shut-off of IFN responses. Such factors, including *UL122*, *UL123, US9, UL31* (reviewed in^9^) and *UL145*^10^, are expressed throughout the viral life cycle. Upon lytic HCMV infection of monocyte-derived dendritic cells (moDCs) the cGAS/STING axis is involved in the induction of type I IFN responses^11^ and as such also a target of viral evasion genes, including *UL83*, *UL122* and *UL138*, whereas the latter is also important for the re-purposing of STAT1 for the establishment of latency^12^. Furthermore, HCMV virions contain proteins, viral coding and non-coding RNAs as well as host mRNAs that can potentially influence host cells directly upon viral entry^13–15^.

In the past, most studies addressed HCMV mediated immune evasion in highly permissive fibroblast cell lines^16,17^. Recently, more work was performed in primary cells such as myeloid cells^17–19^, although under in vitro conditions myeloid cells are not particularly susceptible to HCMV infection when compared with fibroblasts. Single-cell RNA sequencing (scRNA-seq) analysis of CD34^+^ stem cell-derived macrophages provided information about the complex expression kinetics of viral genes^18^ and revealed that permissiveness was not determined by viral entry^17,18^. Recently, a study showed that the intrinsic, but not virus-induced levels of ISG expression are critical for the infection outcome in macrophages^19^.

Here, we aimed to uncover factors that facilitate productive HCMV infection of moDCs. To this end, we performed scRNA-seq analysis of HCMV exposed moDCs. In-depth analysis of virion-associated RNAs confirmed that upon moDC exposure to HCMV most cells got infected, whereas only few cells supported viral gene expression. Most cells that initiated viral immediate early (IE) gene expression progressed to productive infection and released HCMV progeny. We found that initiation of IE viral gene expression correlated with *IFNB1* expression, and that STING induction increased the percentage of IE expressing moDCs. Upon progression of HCMV infection, *IFNB1* but not *IFNL1* expression was inhibited, and ISG expression was shut off. Furthermore, we identified pro- and anti-viral host genes that were associated with increased HCMV gene expression in moDCs.

## Results

### Single-cell RNA sequencing indicates distinct clusters amongst HCMV-NG exposed moDCs

**Fig. 1:**
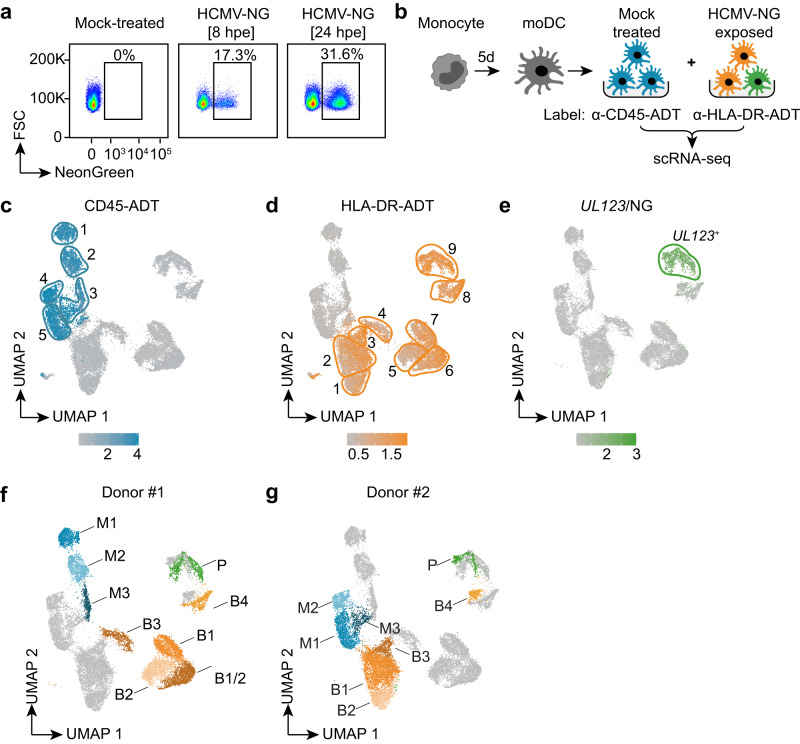
Single-cell RNA sequencing reveals heterogeneity of human monocyte-derived dendritic cells. Blood-derived CD14^+^ monocytes were differentiated to moDCs and exposed to HCMV-NG (NeonGreen) at MOI 6. **a** Flow cytometry analysis of mock-treated and HCMV-NG exposed moDCs 8 and 24 hours post virus exposure (hpe). **b** Schematic depiction of the experimental setup (symbols from BioRender). Mock-treated and HCMV-NG exposed moDCs were labeled 8 hpe with anti-CD45-ADT and anti-HLA-DR-ADT antibodies, respectively, and pooled prior to scRNA-seq. This experiment was performed in four runs with moDCs from two donors and with two independent virus preparations. **c**, **d** Data from all four scRNA-seq runs were combined for non-linear dimensionality reduction (UMAP) and unsupervised clustering (bordered and numbered areas). Log normalized feature counts are shown for CD45-ADT (cells shown in blue) (**c**) and HLA-DR-ADT (cells shown in orange) (**d**). Two clusters (shown without borders) were identified to be composed of doublets and were removed for further analysis. **e** UMAP visualization of SCTransform normalized feature counts for the viral *UL123*/*NeonGreen* gene (cells shown in green). **f** Donor #1 and **g** donor #2 comprised 3 mock treated clusters (M1-3, CD45-ADT^+^), 4-5 bystander clusters (B1-4, HLA-DR-ADT^+^/*UL123*^low^), and 1 productively infected cluster (P, HLA-DR-ADT^+^/*UL123*^high^). Different colors show clusters with divergent gene expression profiles between mock, bystander, and productively infected moDCs from the two different analyzed donors.

To investigate myeloid cell responses to HCMV infection, monocytes were isolated from the blood of healthy donors and cultured for 5 days in medium supplemented with GM-CSF and IL-4 to differentiate moDCs. Such cells showed reduced expression of the monocyte/macrophage marker CD14 and enhanced expression of the DC marker CD209 (Supplementary Fig. 1a), highlighting that the applied DC differentiation protocol was suitable for generating moDCs. Upon exposure of moDCs to the HCMV reporter strain HCMV TB40/E-UL122/123-mNeonGreen (HCMV-NG), that expresses NG at an equimolar ratio with the IE1/IE2 (UL122/UL123) protein^20^, only a minor part of the cells showed NG expression (Fig. 1a). To address whether certain transcriptomic profiles determined HCMV permissiveness of moDCs, we studied the heterogeneity of moDCs exposed to HCMV-NG after 8 h of incubation by scRNA-seq. To minimize batch effects, mock treated and HCMV-NG exposed cells were labeled with anti-CD45 and anti-HLA-DR antibodies carrying specific DNA oligo tags (antibody-derived tags [ADT]) (Fig. 1b), respectively. These markers were used since CD45 and HLA-DR expression was not negatively affected by HCMV exposure (Supplementary Fig. 1b). After pooling of mock treated and HCMV-NG exposed samples and scRNA-seq analysis, the presence of ADT was used to de-multiplex the cells during data analysis^21^. moDCs from two different donors were HCMV-NG exposed, each with two independently produced HCMV-NG preparations (V1, V2) (Supplementary Fig. 1c, d). Accordingly, data from a total of four separate runs with an overall 18,936 cells that passed quality control were combined, analyzed by unsupervised clustering, and visualized using non-linear dimensionality reduction (Fig. 1c, d). CD45-ADT^+^ (Fig. 1c) and HLA-DR-ADT^+^ cells (Fig. 1d) clustered separately. Furthermore, the samples segregated according to the donor origin of the moDCs, whereas batch effects between runs and the two different virus preparations were minimal (Supplementary Fig. 1d). Amongst HCMV-NG exposed cells, only one cluster showed strong expression of *UL123* (Fig. 1e). This cluster also showed an overall higher content of viral RNAs (Supplementary Fig. 1e). Thus, the *UL123* high moDC cluster we assumed to be “productively infected” (P) (Fig. 1e, f, g), whereas cells that were HCMV-NG exposed but did not show strong viral gene expression we termed “bystander” cells (B) (Fig. 1d, f, g), and cells that were not exposed to the virus we termed “mock treated” (M) (Fig. 1c, f, g). For further analysis, single clusters that were composed of cells from both donors were manually split according to the donor origin. As a result, moDCs of each donor comprised three mock treated clusters (CD45-ADT^+^: M1, M2, M3), four to five bystander clusters (HLA-DR-ADT^+^/*UL123*^low^: B1, B1/2, B2, B3, B4), and one productively infected cluster (HLA-DR-ADT^+^/*UL123*^high^: P) (Fig. 1f, g and Supplementary Data File 1).

### Most HCMV-NG exposed moDCs contain virion-associated RNAs, but only a few ones show de novo viral gene expression

**Fig. 2:**
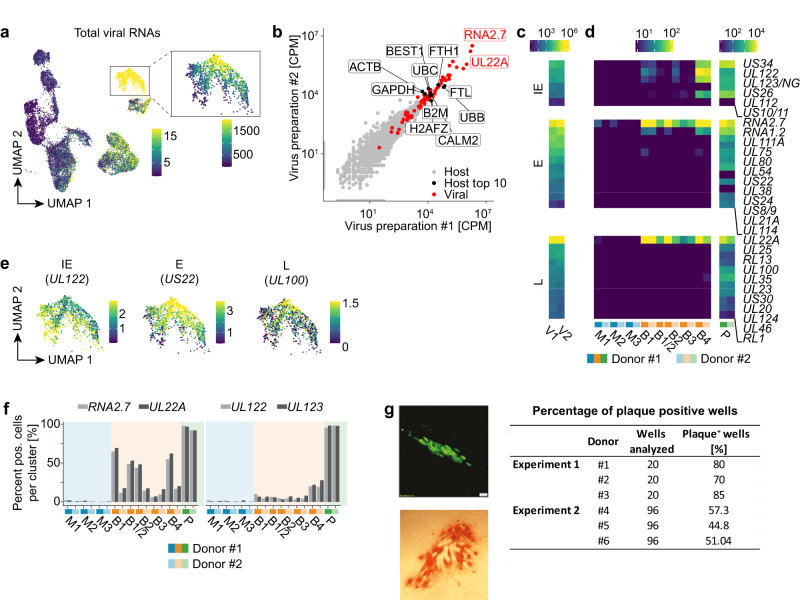
Most HCMV-NG exposed moDCs contain virion-associated RNAs, but only few ones show de novo viral gene expression. **a** UMAP embedding showing the total expression of viral RNAs (SCTransform normalized values) in the dataset analyzed also in Fig. 1. The color scale is chosen to highlight cells with weak viral gene expression, and all cells shown in yellow have expression values > 15. The inset shows the total expression of viral RNAs for cluster P with a different color scale resolving cells with strong viral gene expression. Here all cells shown in yellow have expression values > 1500. **b** Scatter plot showing the abundance of host (grey dots) and viral (red dots) RNAs (in counts per million) in the two independent virus preparations. The two most abundant viral RNAs (*RNA2.7, UL22A*) and the ten most abundant host RNAs (black dots) are labeled. Heatmap showing the abundance of viral RNAs in the virus preparations V1 and V2 (**c**) and in the clusters detected by scRNA-seq (**d**) (counts per million). IE genes are significantly more expressed in B4 than in B1-3 (p < 3.5e-57, two-sided Wilcoxon rank sum test). **e** UMAP embeddings depicting the expression (SCTransform normalized values) of representative viral RNAs of the different kinetic classes of HCMV gene expression in P. **f** Bar diagram showing the abundance (% of all cells that have detectable expression) of virion-associated (*RNA2.7 and UL22A*) and de novo transcribed (*UL122 and UL123*) viral RNAs in mock-treated and HCMV-NG exposed cells. **g** HCMV-NG exposed moDCs were FACS sorted and single NG^+^ moDCs were seeded on monolayers of MRC-5 cells. After 4 days of incubation, NG^+^ plaques were detected in the MRC-5 monolayer by wide-field fluorescent microscopy. A representative NG^+^ plaque is shown in the upper panel, and in the lower panel, one representative IE1^+^ plaque is shown, as detected by light microscopy of HRP-IE1 immunolabeled cells. The table shows the quantification of the number of wells analyzed and the number of wells showing NG^+^ plaques from 6 independent donors from 2 independent experiments.

Many HCMV genes share one polyadenylation site. As 3’ sequencing-based scRNA-seq cannot distinguish such genes, we manually curated groups of viral RNAs belonging to one polyadenylation site (Supplementary Fig. 2a). Analysis of viral RNAs revealed that most HCMV-NG exposed moDCs contained at least trace amounts of viral RNAs (Fig. 2a and Supplementary Fig. 1e). To distinguish virion-associated RNAs that were packaged into virus particles during the process of virus formation, that were delivered to cells by the infection process, and the viral RNAs that were de novo transcribed in infected cells, we sequenced the two HCMV-NG preparations V1 and V2 that we used in the infection experiments. In these sequences in addition to viral RNAs we detected several thousands of different host RNAs (Fig. 2b). The four most abundant host RNAs included the heavy and light subunit of iron storage protein ferritin (*FTL, FTH1*) and the two polyubiquitin genes *UBB* and *UBC* (Fig. 2b). Moreover, we confirmed the presence of the late (L) viral transcript *UL22A* and the early (E) long non-coding *RNA2.7* as the most abundant virion-associated RNAs (Fig. 2c and Supplementary Fig. 2b)^22^. Indeed, these RNAs were also the most abundant ones in bystander moDCs upon exposure to HCMV-NG (Fig. 2d and Supplementary Fig. 2b), indicating that bystander moDCs were entered by HCMV-NG virions and these released virion-associated *UL22A* and *RNA2.7*. In contrast, presumably productively infected cells in P (*UL123*^high^) showed broad expression of viral RNAs of all kinetic classes already at 8 hpe, including IE, E, and L genes (Fig. 2d, e and Supplementary Fig. 2b). In fibroblasts, higher numbers of viral particles entering a single cell correlate with faster progression of the viral life cycle^23^. Interestingly, also in HCMV-NG exposed moDCs we found that the expression of E (e.g., *US22*) and L (e.g., *UL100*) RNAs (Fig. 2d, e) correlated with higher loads of total viral RNAs (Fig. 2a) as well as with the abundance of virion-associated RNAs *UL22A* and *RNA2.7* (Supplementary Fig. 2c). We further addressed simultaneous expression of all classes of viral genes by flow cytometry of moDCs that were exposed to the HCMV^3F^ reporter virus^24^. This virus expressed three different fluorescent proteins, mNeonGreen (NG), mTag blue fluorescent protein 2 (BFP), and mCherry together with IE, E, and L viral genes, respectively. In accordance with the scRNA-seq data, similar percentages of HCMV^3F^-exposed moDCs showed NG, BFP, and mCherry expression (Supplementary Fig. 2d). These results indicated that most of the cells that show IE gene expression progressed to L viral gene expression.

To estimate for each cluster the percentage of cells that carried virion-associated RNAs as compared to the percentage of cells that started de novo viral gene expression, we analyzed the abundance of *UL22A/RNA2.7* versus *UL122/UL123* as prototypic virion*-*associated and de novo expressed RNAs, respectively. This analysis confirmed that close to 100% of cells in P were infected and started viral gene expression (Fig. 2f). Importantly, up to 70% of the bystander cells in B1-3 also showed detectable levels of the virion-associated RNAs *UL22A* and *RNA2.7* (Fig. 2f, left panel). These percentages were presumably even underestimated due to RNA degradation during the 8 h period of incubation and some variation of values was detected between clusters and donors. In contrast, only 5-10% of cells in B1-3 and 25% of cells in B4 showed expression of *UL122* and *UL123* (Fig. 2f, right panel). These data indicate that upon HCMV-NG exposure, most moDCs are entered by virus particles that deliver viral and human RNAs into the cells, whereas de novo viral gene expression is initiated only in a minor fraction of the moDCs. This notion was supported by sorting of *UL122/123*-NG^−^ and *UL122/123*-NG^+^ moDCs at 24 hpe (Supplementary Fig. 3a, b). Upon separate incubation of *UL122/123*-NG^−^ and *UL122/123*-NG^+^ cells in fresh medium for 24 h, some of the *UL122/123*-NG^−^ cells again initiated NG expression suggesting that *UL122/123*-NG^−^ cells initially had been entered by virus particles and were able to reinitiate viral gene expression in the absence of *UL122/123*-NG^+^ cells.

The bystander cluster, B4, showed a viral gene expression profile that was distinct from all other bystander clusters and that included several IE genes (Fig. 2c, Supplementary Fig. 2b). Of note, cells in B4 expressed considerably higher levels of IE genes than cells in the other B clusters (Fig. 2d, f) and in the UMAP B4 was placed relatively close to P (Fig. 1d, e). Nonetheless, RNA velocity analysis^25^ did not reveal the transition of B4 cells to productively infected cells in P (Supplementary Fig. 3c), suggesting that cells in B4 were abortively or latently infected.

To determine whether all moDCs in which high UL123 expression is initiated progress to productive infection we sorted and seeded single *UL122/123*-NG^+^ moDCs in separate wells containing each a monolayer of MRC-5 cells. 65% of the single *UL122/123*-NG^+^ moDCs produced live viral progeny, as indicated by the formation of HCMV plaques in the MRC-5 monolayer (Fig. 2g).

These data showed that upon HCMV exposure the majority of moDCs get infected, whereas viral gene expression is supported only by some cells, of which a large extent progresses to productive infection.

### Increased expression of viral RNAs is associated with the downregulation of interferon-stimulated genes and upregulation of heat shock proteins

**Fig. 3:**
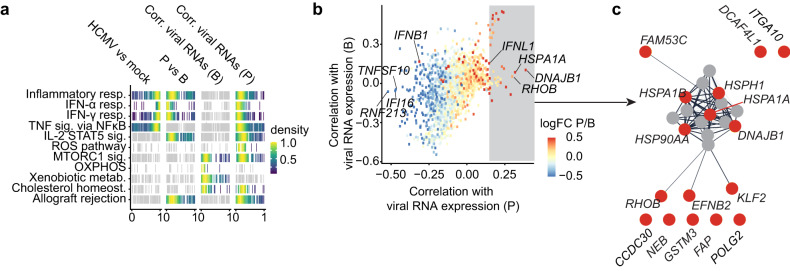
Viral RNA expression is associated with decreased ISG and increased heat shock protein expression. **a** Pathway analysis (i) of differentially regulated genes in HCMV-NG exposed versus mock-treated (1st panel), (ii) of productively infected versus bystander moDCs (2nd panel), and (iii) correlation of host genes with viral RNA expression in bystander (3rd panel) and (iv) in productively infected moDCs (4th panel). Shown are all MSigDB Hallmark pathways in which at least one analysis was statistically significant (highlighted in color, *p* < 0.01, two-sided Wilcoxon test, Benjamini-Hochberg multiple testing correction). Each vertical line is the rank of the fold change (1st and 2nd panel) or of the Spearman correlation (3rd and 4th panel) for a pathway gene. Ranks are divided by the total number of genes in a manner that rank 0 represents the value of the most down-regulated (1st and 2nd panel) or negatively correlated genes (3rd and 4th panel), whereas rank 1 represents the most up-regulated (1st and 2nd panel) or positively correlated (3rd and 4th panel) gene. Colors represent kernel density estimates of ranks with the mode of the density scaled to 1. **b** Spearman’s correlation coefficients between total viral RNA expression and expression of individual host genes across bystander cells (B, y-axis) and productively infected cells (P, x-axis). Dots are colored depending on their expression level in P relative to B (FC, fold change) revealing more down- (blue, *n* = 94) than upregulated (red, *n* = 29) genes (Wilcoxon test, false discovery rate < 0.01, absolute log_2_ fold change >0.5). **c** Protein-protein interaction network derived from the functional enrichment analysis provided by the STRING database. The data shows the 16 most positively correlated genes in HCMV-NG exposed clusters (highlighted in red). Connections represent predicted functional evidence for protein-protein interactions.

Hallmark pathway analysis indicated that genes from inflammatory pathways were significantly enriched among genes that were upregulated in moDCs upon HCMV-NG exposure when compared with mock-treated cells (Fig. 3a, first panel, two-sided Wilcoxon test comparing the distribution of log_2_ fold changes of pathway genes vs. all other genes). Strikingly, genes from inflammatory pathways were significantly enriched among downregulated genes in productively infected cells in P when compared with bystander cells in B1-4 (Fig. 3a, second panel, two-sided Wilcoxon test). Moreover, in bystander cells, genes assigned to homeostatic/metabolic pathways were significantly enriched among genes that were negatively correlated with viral gene expression (Fig. 3a, third panel, two-sided Wilcoxon test comparing the distribution of correlation coefficients with viral gene expression of pathway genes vs. all other genes), whereas in productively infected cells, genes assigned to inflammatory and interferon response pathways were enriched among negatively correlated genes (Fig. 3a, fourth panel, two-sided Wilcoxon test). Thus, the downregulation of host response pathways to infection was qualitatively and quantitatively dependent on viral gene expression.

Interestingly, more host genes were downregulated (*n* = 94, Wilcoxon test, false discovery rate <0.01, absolute log2 fold change >0.5) than upregulated (*n* = 29) in productively infected cells (P) when compared with bystander cells (B) (Supplementary Data File 2). Downregulated host genes were mostly negatively correlated with the expression of viral RNAs and comprised predominantly ISGs (Fig. 3b). Indeed, we identified three ISGs that correlated most negatively with host genes in P, including *IFI16*, *RNF213*, and *TNFSF10* (TRAIL). Of note, some markers were positively correlated with both bystander and productively infected cells (Fig. 3b, upper right corner). Nonetheless, in-depth analysis revealed that these genes were donor-dependent and not a common phenotype shared between different donors.

In contrast, in the few cases where host genes were upregulated in productively infected cells (P), they strongly positively correlated with viral gene expression (Fig. 3b). This included the GTP-binding protein RhoB and a broad range of heat shock proteins (HSPs) (Fig. 3b, c). In particular, the HSPs *DNAJB1* and *HSPA1A* were the host genes that were most positively correlated with viral RNA expression in P. Thus, here we identified putative pro- and anti-viral host factors of HCMV infection in moDCs. Interestingly, also *IFNL1* (the gene encoding IFN-λ1) was one of the few host genes that was upregulated in P and that was positively correlated with viral gene expression. In contrast, *IFNB1* (the gene encoding IFN-β) was upregulated in P, and in contrast to all other upregulated genes, *IFNB1* correlated negatively with viral gene expression (Fig. 3b). This indicates that IFNB1 was induced in infected cells, but this induction was blunted in cells with strong viral gene expression.

### Viral IE gene expression is correlated with *IFNB1* expression, whereas progression of viral infection inhibits *IFNB1*, but not *IFNL1* induction

**Fig. 4:**
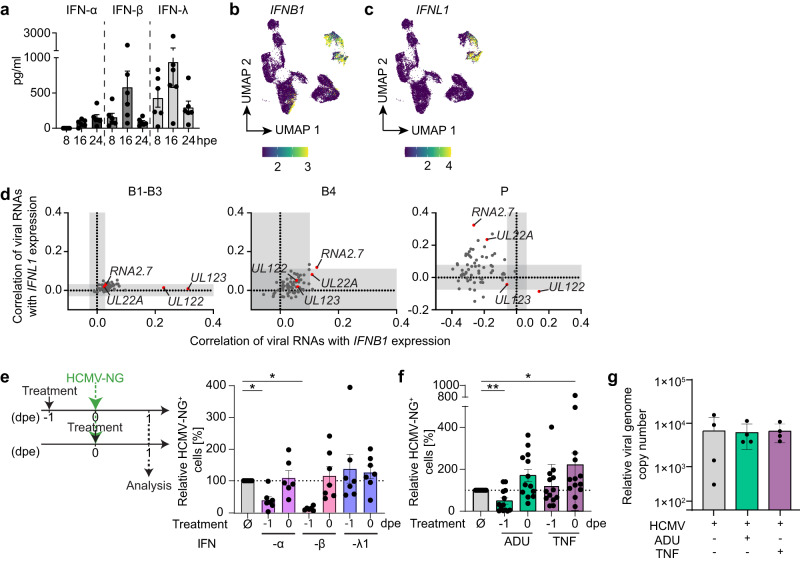
*IFNB1* correlates with viral IE gene expression, whereas the progression of viral infection inhibits *IFNB1*, but not *IFNL1* induction. **a** moDCs were exposed to HCMV-NG and supernatants were harvested completely and replenished with fresh medium 8, 16 and 24 hpe to determine the IFN-α, IFN-β or IFN-λ content by ELISA methods. Mean ± SEM of 6 different donors from 2 independent experiments. UMAP embeddings showing expression levels (SCTransform normalized values) of *IFNB1* (**b**) and *IFNL1* (**c**). **d** Spearman´s correlation coefficients of each individual viral gene with *IFNB1* (x-axis) and *IFNL1* (y-axis) expression for clusters B1-3 (1st graph), B4 (2nd graph), and P (3rd graph). White areas indicate statistically significant regions (*p* < 0.01, approximate two-sided t-test, Benjamini-Hochberg multiple testing correction). moDCs were treated one day before HCMV-NG exposure (−1) or at the time of HCMV-NG exposure (0) with **e** IFN-α2b (lavender bars), IFN-β (pink bars), or IFN-λ1 (blue bars), and **f** ADU-S100 **(**ADU) (green bars), or tumor necrosis factor (TNF) (purple bars), and NG^+^ cells were quantified by flow cytometry one (1) day post HCMV-NG exposure (dpe). Values for NG^+^ cells after HCMV-NG exposure and the treatment as indicated are shown relative to values for NG^+^ cells after HCMV-NG exposure without any other treatment, which were set to 100%. Values for HCMV-NG exposed cells without any other treatment are the same in **e** and **f** as the experiments were performed simultaneously. **e** Mean ± SEM of 7 different donors from 3 independent experiments. Each dot represents a single donor. *p = 0.0156 (−1 IFN-α vs. untreated), *p* = 0.0313 (−1 IFN-β vs. untreated) using two-sided paired Wilcoxon signed-rank test. (f) Mean ± SEM of 13 different donors from 6 independent experiments. *p = 0.0266, **p = 0.0078 using two-sided paired Wilcoxon signed-rank test. **g** M1, M2 and M3 moDC subsets were FACS sorted and left untreated (grey bar) or treated with ADU-S100 (ADU) or tumor necrosis factor (TNF) (green and purple bar, respectively) followed by exposure to HCMV-NG for 4 h. The genomic DNA was extracted from the soluble nuclear fraction and the relative abundance of HCMV genomes in relation to the housekeeping gene GAPDH was analyzed by qPCR. Mean ± SEM of 4 different donors from 1 experiment.

To further study the regulation of different interferons during infection, we exposed moDCs to replication-competent HCMV-NG and UV-inactivated HCMV-NG and analyzed cell-free supernatants for the presence of IFN-α, IFN-β, and IFN-λ. Both IFN-β and IFN-λ were mostly secreted within the first 16 hpe, whereas IFN-α secretion started later (Fig. 4a and Supplementary Fig. 4a). Accordingly, mainly *IFNB1* and *IFNL1* expression was detected in the scRNA-seq analysis at 8 hpe (Fig. 4b, c). *IFNB1* was expressed in some cells of B1-3, B4 and P (Fig. 4b), whereas *IFNL1* expression was restricted mainly to B4 and some cells in P (Fig. 4c). While *IFNL1* expression was also positively correlated with total viral RNA levels in P, *IFNB1* was negatively correlated, suggesting a different mechanism of regulation for *IFNB1* and *IFNL1* (Fig. 3b and Supplementary Fig. 4b). Therefore, we performed a detailed correlation of viral and host genes with *IFNB1* and *IFNL1* expression. In B1-B3, both *IFNB1* and *IFNL1* showed overall low correlations with virtually all viral and host genes (Fig. 4d, Supplementary Fig. 4c and Supplementary Data File 3 and 4), including correlations with the most abundant viral RNAs in B1-B3, i.e., the virion-associated RNAs *UL22A/RNA2.7*. Notably, the only exceptions were the viral IE genes *UL122* and *UL123*, which showed a high correlation exclusively with *IFNB1* in B1-3 (Fig. 4d left panel). In B4, *IFNB1* and *IFNL1* did not show a significant correlation with most viral genes (Fig. 4d middle panel), whereas a large number of host genes was strongly correlated with both IFNs (Supplementary Fig. 4c). In particular, *PPP1R15A* (GADD34) was highly positively correlated with both IFNs in B4. *PPP1R15A* was also positively correlated with both IFNs in productively infected cells of P. In P *IFNB1* expression was positively correlated only with the viral IE gene *UL122*, while it was negatively correlated with all other viral RNAs (Fig. 4d right panel) suggesting that upon progression to E and L stages, *IFNB1* expression was counter-regulated. In stark contrast, *IFNL1* was positively correlated with the majority of viral RNAs in P, in particular with *UL22A* and *RNA2.7* (Fig. 4d right panel).

To address the anti-viral activity of the different IFNs produced by HCMV-NG exposed moDCs, we treated moDCs with IFN-α2b, IFN-β or IFN-λ1 at the time of (0 dpe), or one day prior (−1 dpe) to HCMV-NG exposure. Treatment of moDCs with these cytokines at the time of virus exposure did not change the percentage of HCMV-NG^+^ cells when compared with infected cells without the cytokine treatment (Fig. 4e). In contrast, pre-treatment with IFN-α2b and IFN-β one day prior to HCMV-NG exposure significantly reduced the percentage of NG^+^ cells (Fig. 4e), whereas pre-treatment with IFN-λ1 did not (Fig. 4e), highlighting the anti-viral effect of IFN-α and IFN-β, but not of IFN-λ1, under such conditions.

Thus, especially the host gene *PPP1R15A* seems to play an important role in the induction of both *IFNB1* and *IFNL1* in moDCs. Moreover, in bystander cells, *IFNB1* expression was associated with viral IE gene transcription, whereas in productively infected cells expression of the highly anti-viral *IFNB1*, but not *IFNL1*, was counter-regulated upon progression of HCMV infection.

### Upon HCMV exposure, STING induction facilitates IE viral gene expression

cGAS/STING recognition of HCMV DNA leads to type I IFN expression in moDCs via interferon regulatory factors (IRFs)^11^. STING also activates NF-κB signaling^26^ and NF-κB transactivates the HCMV major IE promoter (MIEP)^26,27^. We, therefore, hypothesized that upon entry of HCMV, cGAS/STING not only induced *IFNB1* expression but also activated HCMV IE gene transcription, thus inducing both *IFNB1* and *UL122/UL123* expression in bystander cells (Fig. 4d). In line with this hypothesis, pre-treatment with the STING agonist ADU-S100 decreased the susceptibility of moDCs to HCMV-NG infection, whereas treatment at the time of virus exposure significantly increased percentages of NG^+^ moDCs (Fig. 4f). Treatment at time of virus exposure with TNF that activates NF-κB, similarly increased HCMV-NG infection. Interestingly, increased HCMV infection upon activation of STING or stimulation with TNF at the time of virus exposure was also observed in other cells of the myeloid lineage such as macrophages (Supplementary Fig. 4d). Thus, STING induction and TNF-mediated NF-κB activation enhanced HCMV IE gene expression in myeloid cells.

STING was recently suggested to facilitate the transport of HCMV genomes into the nucleus^28,29^. Thus, we next sought to test whether the virus DNA enters the nucleus of moDCs to different extents. Interestingly, treatment with STING or NF-κB agonists at the time of virus exposure did not affect the efficiency of nuclear delivery of the viral genome (Fig. 4g), suggesting that the increase in HCMV infection upon STING induction was not mediated by the increased nuclear import of HCMV genomes.

### moDCs show a marked downregulation of ISGs upon progression to productive infection

**Fig. 5:**
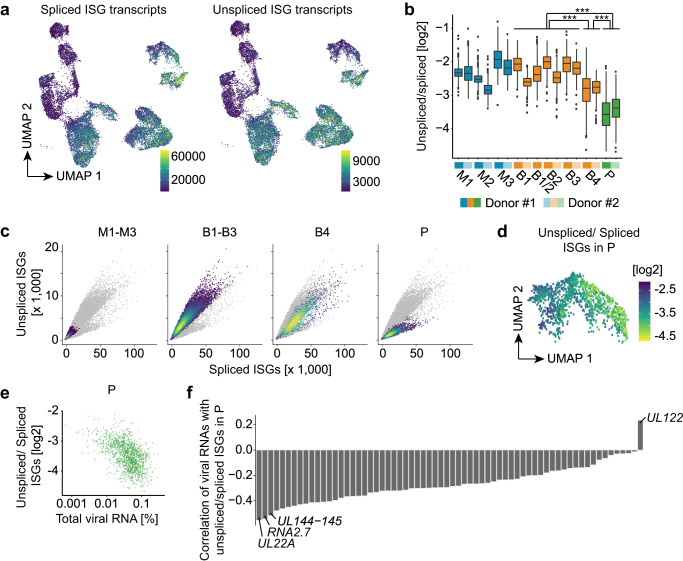
Initial ISG transcription in productively infected cells is inhibited upon efficient viral gene expression. **a** UMAP embeddings highlighting the expression (raw feature counts) of spliced and unspliced ISG RNAs (total sum of all ISGs as defined by MSigDB). **b** Boxplots showing the distribution of unspliced vs. spliced log_2_ fold changes for ISG RNAs in all clusters (***p < 2.22e−16, two-sided Wilcoxon ranks-sum test; center line, median; box limits, upper and lower quartiles; whiskers, 1.5x interquartile range; points, outliers). Log_2_ fold changes are computed from the values shown in panel **a**. **c** Phase portraits showing expression of unspliced (y-axis) and spliced (x-axis) ISG RNAs per cell. Cells from clusters M1-3 (1st graph), B1-3 (2nd graph), B4 (3rd graph), and P (4th graph) are highlighted in color. **d** UMAP depicting the ratio of unspliced/spliced ISG RNAs per cell in P. **e** Unspliced/spliced ISG RNAs in P (y-axis) compared to total viral RNA expression (x-axis, in percentage relative to total feature counts per cell). **f** Spearman’s correlation coefficient for the ratio of unspliced/spliced ISG RNAs with the expression of single viral RNAs in P.

Commonly employed analysis pipelines for scRNA-seq data only consider reads originating from mature RNAs (“spliced reads”). As splicing occurs predominantly co-transcriptionally, the abundance of unspliced reads is a good approximation of nascent RNA that is currently being transcribed in an individual cell. This parameter has previously been used by the RNA velocity method to predict future states of cells^25^. Here, we compared unspliced and spliced RNA to analyze the recent regulation of individual genes. All HCMV-NG exposed moDCs, including cells in P, showed higher levels of spliced ISG reads than mock-treated cells (Fig. 5a, left UMAP) suggesting that ISGs were initially induced in virtually all HCMV-NG exposed cells, including productively infected cells. In constrast, unspliced reads for ISG RNAs were massively reduced in productively infected cells of P when compared with bystander cells (Fig. 5a, right UMAP) suggesting that after the initial ISG induction in B and P, ISG expression was shut off in P. Accordingly, the ratio of unspliced vs. spliced ISG reads was substantially lower in P than in B1-4 (Fig. 5b). A separate analysis of unspliced versus spliced RNAs in M1-3, B1-3, B4, and P confirmed that although all moDCs were competent to express ISGs upon HCMV-NG exposure, cells that contained intermediate amounts of viral RNA such as in B4 moderately downregulated ISG transcription. Moreover, cells with strong viral gene expression in P showed massive downregulation of ISG transcription (Fig. 5c and Supplementary Fig. 5a–c). Notably, a subset of ISGs, including *ZC3HAV1* (ZAP) and *OASL*, were only strongly induced in B4 and P, but not in B1-3 (Supplementary Fig. 5d, e). While their expression in P was inhibited, they showed no inhibition and even much stronger gene expression in B4 than in P. Thus, B4 does not only express a distinct viral gene profile (Fig. 2d, Supplementary Fig. 2b), but also lacks downregulation of a subset of ISGs. Importantly, HCMV does not generally affect host gene splicing as observed for the expression of *B2M* (Supplementary Fig. 5f). In P, the extent of ISG shut-off correlated with overall levels of viral gene expression (Fig. 5d, e). Interestingly, active ISG transcription was positively correlated with *UL122*, but negatively correlated with all other viral RNAs, in particular *UL144-UL145*, *RNA2.7*, and *UL22A* (Fig. 5f). These correlations indicated that massive suppression of ISG expression was initiated after the IE phase of viral infection.

### moDCs comprise three distinct clusters with distinct gene expression profiles

**Fig. 6:**
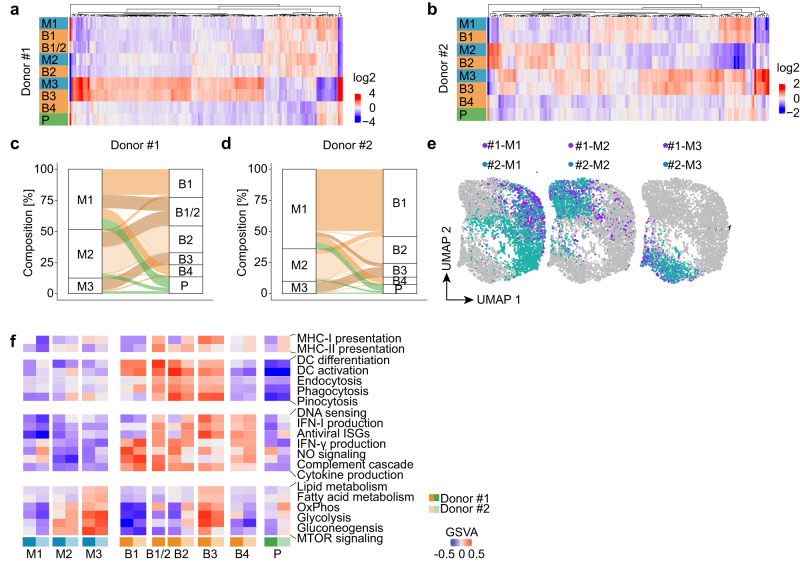
HCMV-NG exposed moDCs can be traced back to three distinct clusters as identified in mock-treated moDCs. Heat maps showing the average expression per cluster (log_2_-fold change vs. the grand mean) of marker genes that were common in mock-treated and HCMV-NG exposed cells of donor #1 (**a**) and donor #2 (**b**). Alluvial charts showing the contribution of mock-treated moDCs to HCMV-NG exposed clusters of donor #1 (**c**) and donor #2 (**d**). **e** Donor integration by canonical correlation analysis of M1, M2, and M3 clusters. The same UMAP embedding is shown for each of the three mock-treated clusters with donors highlighted. **f** Pathway analysis of manually selected DC characteristics pathways for the different clusters using gene set variation analysis (GSVA). Positive GSVA scores indicate an enrichment of strongly expressed genes in a pathway.

Since mock-treated moDCs of each donor separated into 3 clusters (M1-3), we next sought to determine whether productively infected cells predominantly originated from one of these clusters. Cluster-specific marker gene expression profiles indicated that for cells from both donors each bystander cluster predominantly originated from a single mock treated cluster, i.e., B1 originated from M1, B2 from M2, and B3 from M3 (Fig. 6a, b and Supplementary Fig. 6a). For a more unbiased analysis, we deployed canonical correlation analysis (CCA) to compute a joint embedding of mock-treated and bystander cells, which removed the mock/bystander effect from data. This embedding was then used to predict for each HCMV-NG exposed cell the most similar mock treated cells, providing further evidence for the origins of clusters B1, B2, and B3 from clusters M1, M2, and M3 as indicated by the marker gene analysis above (Fig. 6c, d). The bystander cluster B1/2 from donor #1 originated from M1 and M2. Interestingly, bystander cells in cluster B4, and productively infected cells in P, were composed of cells from all three mock-treated clusters. Importantly, M1-derived cells were the most abundant ones in cluster P (Fig. 6c, d), even after normalizing for the different cluster sizes of M1-M3 (Supplementary Fig. 6b, c) suggesting that M1 cells showed the highest susceptibility to HCMV infection.

Comparing cells from both donors using CCA, as introduced above, revealed strong similarities between the M1 cluster of donor 1 and the M1 cluster of donor 2, the M2 cluster of donor 1 and the M2 cluster of donor 2 and the M3 cluster of donor 1 and the M3 cluster of donor 2 (Fig. 6e). This similarly applied for their respective HCMV-NG exposed counterparts (Supplementary Fig. 6d). To address whether moDC clusters M1-3 represented distinct subsets of moDCs or different stages of differentiation, we performed RNA velocity analysis that predicts the upcoming gene expression profile of cells. This analysis did not reveal any particular directionality among the clusters M1-3 (Supplementary Fig. 6e). Instead, pathway analysis of moDC-specific traits revealed that the clusters M1-3 showed differences in metabolic pathways, cytokine production, endocytosis, and in their antigen presentation capacity (Fig. 6f, Supplementary Fig. 6f and Supplementary Data File 5). Notably, the respective clusters from donors #1 and #2 showed overall very similar profiles of active pathways. Taken together, moDCs comprise three distinct clusters with distinct gene expression profiles that were similarly detected amongst mock-treated and HCMV-NG exposed moDCs derived from two independent donors.

### The three moDC clusters are defined by characteristic protein expression profiles and differential susceptibilities to HCMV infection

**Fig. 7:**
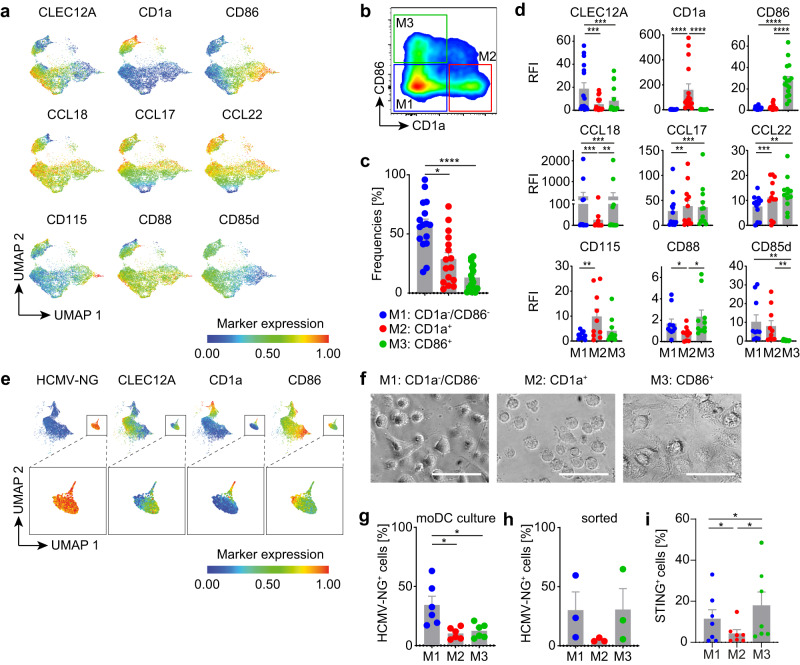
moDCs comprise three different subsets that are defined by characteristic protein expression profiles and differential susceptibilities to HCMV infection. moDCs were differentiated from 16 independent donors, CLEC12A, CD1a, CD86, CCL18, CCL17, CCL22, CD115, CD88 and CD85d were immunolabeled and analyzed by flow cytometry. **a** UMAP of live cells showing heatmap coloring to indicate the abundance of each of the immunolabeling markers, data of one representative donor are shown. **b** Mock-treated moDCs were immunolabeled as described above and the three moDC subsets were discriminated by gating of CD1a^−^/CD86^−^ (M1, blue dots), CD1a^+^ (M2, red dots) and CD86^+^ (M3, green dots) cells. **c** Frequencies and **d** relative fluorescence intensities (RFI) of each of the analyzed markers in the three subset gates were determined. Data represents mean ± SEM of 16 different donors from 7 independent experiments. Each dot represents a single donor. ****p < 0.0001, *p = 0.0182 using two-sided paired Wilcoxon signed-rank test (**c**) and ****p < 0.0001 (CD1a, CD86), ***p = 0.0010 (CCL17, CCL18), *p* = 0.0003 (CLEC12A), *p* = 0.0005 (CCL18, CCL22), **p = 0.0039 (CD115), *p* = 0.0093 (CCL18), 0.0020 (CD85d), *p* = 0.0015 (CCL17, CCL22), *p = 0.0137 (CD88) using two-sided paired Wilcoxon signed-rank test (**d**). **e** moDCs were infected with HCMV-NG, immunolabeled and analyzed as in **a**. UMAP of NG fluorescence and the 3 most discriminative moDC subset markers, i.e., CD1a, CD86, and CLEC12A. Insets show the expression of the above-mentioned markers only in the NG^+^ cluster. **f** Mock-treated moDC subsets were sorted using the gating strategy shown in (**b**) and analyzed morphologically (scale bar 50 µm). moDC cultures (**g**) or sorted moDCs (**h**) as described in **b** were infected with HCMV-NG and percentages of NG^+^ cells were determined 24 hpe. Data represents mean ± SEM of 6 different donors from 3 independent experiments. Each dot represents a single donor. *p = 0.0313 using two-sided paired Wilcoxon signed-rank test (**g**) and ± SEM of 3 different donors from 1 experiment. Each dot represents a single donor. **i** Quantification of STING protein expression from the subsets described in **b**. Data represents mean ± SEM of 7 different donors from 4 independent experiments. Each dot represents a single donor. *p = 0.0469 (M2 vs. M1), *p* = 0.0156 (M3 vs. M2), and *p* = 0.0313 (M3 vs. M1) using two-sided paired Wilcoxon signed-rank test.

Next, we verified by flow cytometry that mock-treated moDCs showed similar protein expression profiles as detected in the moDC clusters using cluster-specific genes that we manually selected from the scRNA-seq data set, i.e., *CLEC12A* (CD371), *CD1a, CD86, CCL18, CCL17, CCL22*, *CSF1R* (CD115), *C5AR1* (CD88), and *LILRB2* (CD85d) (Fig. 7a–d, Supplementary Fig. 7a, b). In particular, the expression of CD1a and CD86 distinguished the 3 subsets (Fig. 7b). M1 was characterized by CD1a^−^/CD86^−^, while showing high expression levels of CLEC12A. CD1a^+^ expression defined M2 and CD86^+^ expression defined M3. Analysis of moDCs from sixteen different donors verified the presence of three subsets and indicated that CD1a^−^/CD86^−^ cells represented approximately 57%, CD1a^+^ 28%, and CD86^+^ 13% of moDCs (Fig. 7c). Furthermore, CLEC12A, CD1a and CD86 discriminated the three subsets also in HCMV-NG exposed moDCs (Fig. 7e). Moreover, the three moDC subsets showed distinct morphologies (Fig. 7f). Higher percentages of NG^+^ cells were detected amongst CD1a^−^/CD86^−^ cells (M1) than amongst CD86^+^ (M3) and CD1a^+^ cells (M2) (Fig. 7g), which is consistent with our scRNA-seq data (Fig. 6c, d). Upon cell sorting of the three moDC subsets and HCMV-NG exposure, the CD1a^−^/CD86^−^ subset (M1) again showed higher infection susceptibility than CD1a^+^ cells (M2) (Fig. 7h). Sorted CD86^+^ cells (M3) showed higher susceptibility to infection than in the mixed moDC cultures that was reminiscent of the infection susceptibility of sorted CD1a^−^/CD86^−^ cells (M1), which presumably was due to the absence of competitive effects contributed by the other moDC subsets in the sorted cultures.

Interestingly, while the overall RNA expression of STING was rather low in moDCs (Supplementary Fig. 7c), protein analysis by flow cytometry of the three moDC subsets revealed that intrinsic levels of STING (in mock treated cells) were lower in M2 than in M1 and M3 moDCs (Fig. 7i and Supplementary Fig. 7d), which correlated with the susceptibility of the three moDC subsets to HCMV infection (Fig. 7h), highlighting a potential link between intrinsic levels of STING expression and the start of IE gene expression. Finally, we sought to understand whether the increase in IE viral gene expression was dependent on the nuclear import of viral genomes upon STING induction. However, no differences were detected in terms of genomic HCMV DNA levels in the nucleus of the three moDC subsets, irrespective of whether the cells were treated with STING or NF-κB agonists (Supplementary Fig. 7e).

Thus, flow cytometry validated the existence of three subsets amongst moDCs that showed different protein expression profiles, including the abundance of STING, and different susceptibilities to HCMV infection, as suggested by scRNA-seq.

## Discussion

Analysis of scRNA-seq data from HCMV-NG exposed moDCs and validation of key findings on the protein level revealed a highly intricate relationship between HCMV gene expression, IFN induction and ISG expression that determines the outcome of HCMV infection of moDCs. We propose the model that upon virus encounter, the majority of moDCs get infected by the virus. Predominantly cells that are entered by a high number of virus particles sense HCMV via the cGAS/STING axis, which leads to the induction of type I IFN and STING-mediated activation of viral IE gene expression. IFN signaling then induces the expression of ISGs in all cells. In some cells (B4) where viral gene expression has started, high expression of specific ISGs suppresses the progression to productive infection and augments the induction of *IFNB1*. However, in most cells that show strong viral IE gene expression, the viral infection cycle progresses, and viral immune evasion molecules efficiently inhibit ISG and *IFNB1* expression. Furthermore, we provide evidence that in vitro differentiated moDCs are composed of three distinct subsets that support virus infection to different extents.

Previously, HCMV virions were shown to contain certain viral and host RNAs^13,22^. Analysis of RNAs contained in the two HCMV-NG preparations used in this study revealed the presence of a plethora of virion-associated host RNAs, some of which have already been described earlier and others that to our knowledge were newly identified here. Several of these host genes are highly upregulated at later time points of infection and thus may be translated early on, right after virus entry into the cell, when viral gene expression is not initiated yet^30^. Particularly, the highly abundant heavy (*FTH*) and light chain (*FTL*) of the iron storage molecule ferritin might have functions in newly infected cells, as HCMV replication and the typical “cytomegaly” phenotype of infected cells depend on iron^31^. Similarly, the two polyubiquitin RNAs *UBB* and *UBC* might be advantageous for the virus because HCMV exploits and repurposes the ubiquitin-proteasome system on different levels of its life cycle, including during latency and lytic infection^32^. In our study, free RNAs were not enzymatically removed. Nevertheless, the contribution of RNAs to the detected total pool of virion-associated RNAs is presumably minimal since the most abundant RNAs that we detected were amongst those that are reported to be packaged into HCMV virions^13^. In the past, several studies addressed the relevance of single host molecules in HCMV replication and their potential function as virion-associated RNAs^33^. Nonetheless, few of these studies achieved significant conclusions. Hence there is a general lack of knowledge about functional implications of virion-associated host as well as viral RNAs that needs to be addressed in future research.

HCMV-encoded *RNA2.7* and *UL22A* are reported to be the most abundantly expressed RNAs in late lytic and latent infection as well as being the most prevalent virion-associated RNAs^34,35^. We confirmed the latter and thus used the presence of *RNA2.7* and *UL22A* in moDCs as proxy markers for viral entry. Since most HCMV-NG exposed moDCs contained these viral RNAs, we concluded that the majority of moDCs were entered by HCMV, whereas only some cells supported the initiation and progression of viral gene expression. In support of this notion, we could show that a high percentage of sorted NG^+^ moDCs were able to release viral progeny, which confirmed the productive infection of the corresponding cells. Cells that did not support productive infection presumably were abortively infected, although, it is tempting to speculate that at least a fraction of these cells were latently infected^35^.

Previously, studies in fibroblasts showed that the onset and progression of viral infection is dependent on the number of virions that infected a single cell^17,23^. Here, we found that already by 8 hpe, HCMV-NG exposed moDCs showed all stages of the virus life cycle, including immediate early, early, and late. Since such cells showed the highest level of virion-associated RNAs, including *UL22A* and *RNA2.7*, it is likely that also in our system a faster progression towards virus replication was caused by higher numbers of infecting virions entering a single cell.

We detected several host genes that were strongly correlated with viral RNA expression and that correspondingly might represent pro- and anti-viral factors, which determine the infection outcome. Among such host RNAs, we identified *RHOB*, which was described earlier as a pro-viral factor during HCMV infection of fibroblasts^36^, suggesting that it was also relevant for HCMV replication in moDCs. Several HSPs such as *DNAJB1* and *HSPA1A* showed pro-viral potential and were earlier described to facilitate replication of herpesviruses, including HCMV^37–39^. Interestingly, among potential anti-viral factors, we found *IFI16*, which was previously characterized as being pro-viral in the immediate early and anti-viral in later stages of HCMV infection^40^. Other anti-viral candidates not described yet in the context of HCMV infection include the broadly anti-viral ISG15 interactor *RNF213*^41^ and *TNFSF10*, which encodes the NK cell-activating protein TRAIL^42,43^. The strong downregulation of TRAIL expression early after HCMV infection that we observed here is likely another mechanism to escape NK cell-mediated killing.

The IFN response is critical for controlling HCMV infection. In accordance with some of our earlier studies, we found that not only productively infected but also bystander cells showed IFN production^11^ and infection seemed to be a prerequisite for IFN induction in moDCs^44^. In bystander cells, *IFNB1* expression was associated with *UL122* and *UL123* expression, but not *UL22A* and *RNA2.7*. HCMV-induced type I IFN expression in moDCs is activated via the cGAS/STING axis^11^. Since STING has recently been suggested to mediate the transport of HCMV genomes into the nucleus in fibroblasts^28^, whereas the YES-associated protein (YAP)-dependent downregulation of STING decreases nuclear import of genomes^29^, we investigated the connection of STING and UL122/UL123 expression in moDCs. Indeed, we found that concomitant HCMV-NG infection and STING induction significantly increased the percentage of NG^+^ moDCs, i.e., of moDC that support viral gene expression. However, no increase of HCMV genomes in the nucleus of such moDCs was found. STING induction  does not only lead to the production of type I IFN^45^ but also triggers the NF-κB signaling pathway^26^. Interestingly, the HCMV major immediate early promoter (MIEP) contains 4 NF-κB binding sites, which have been suggested to be important for IE gene expression in non-dividing cells such as human umbilical vein endothelial cells (HUVEC)^46^. In contrast, in latently HCMV-infected CD14^+^ monocytes in which the virus was reactivated by differentiating the cells into moDCs in the presence of IL-6, deletion of a creb response element (CRE) and mitogen and/or stress-activated kinases impaired IE gene expression, but deletion of NF-κB binding sites from MIEP had only a minor effect^47^. Interestingly, in our study, concomitant HCMV-NG infection and TNF mediated NF-κB activation similarly increased the percentage of NG^+^ moDCs as concomitant HCMV-NG infection and STING induction. This is compatible with the hypothesis that upon infection of moDCs with HCMV-NG, STING-dependent NF-κB activation of MIEP might mediate the increase in HCMV IE gene expression. However, this hypothesis needs to be further addressed experimentally, which in future experiments can be done by using an HCMV TB40/E variant in which the 4 NF-κB binding sites have been mutated^47^. Nevertheless, the increase in HCMV gene expression upon STING induction in moDCs is another example of how HCMV, like many other viruses, re-purposes key anti-viral host factors for its own benefit^48^.

Productive HCMV infection rapidly shuts off ISG expression. Remarkably, by analyzing spliced and unspliced ISG RNAs, we found that productively infected cells initially expressed ISGs, which might even have facilitated HCMV infection, as proposed earlier^18^. Interestingly, among the viral RNAs that showed the highest association with the ISG shut off upon progression of infection, we found *UL144-UL145*. *UL145* was previously identified to be essential for the induction of proteasomal degradation of STAT2 in fibroblasts^10^. Together with our data, this suggests that *UL145* is one of the major viral factors that confers efficient ISG shut-off in moDCs.

As moDCs are extensively used in basic research as well as in clinical application^49^, we further addressed the composition of in vitro generated moDCs. A recent paper analyzing scRNA-seq data of moDCs differentiated from a single donor reported the presence of seven different moDC subsets^50^. Here we analyzed moDCs differentiated from monocytes of two donors, which allowed us to discriminate experimental and donor-associated variations from true effects. Correspondingly, we detected three transcriptionally distinct moDC clusters, which were defined by the expression of characteristic surface marker profiles, i.e., subset 1 being CD1a^−^/CD86^−^/CLEC12A^+^, subset 2 being CD1a^+^, and subset 3 being CD86^+^. Importantly, we verified the presence of these three subsets in moDCs derived from a total of sixteen independent donors. While the absence of CD1a expression theoretically could be a result of in vitro differentiation, other publications have shown the existence of a CD1a^−^ DC population, which is referred to as dermal DC^51,52^. Furthermore, CD1a re-expression has been described on moDCs that were depleted of CD1a^+^ cells^53^, suggesting that these cells represent a distinct subset with a defined differentiation status. This data further confirmed earlier studies that showed the existence of CD1a^+^ and CD1a^−^ moDCs^53,54^ and we additionally identified a third subset characterized by the expression of CD86. Interestingly, RNA velocity analysis suggested that the three moDC populations are distinct cell subsets rather than different differentiation stages of the same subset. Importantly, several functions of in vitro derived moDCs were also detected in the putative in vivo counterparts, based on similar expression of cell surface markers, as well as by transcriptional profiling^55^. However, due to their ontogeny, the populations identified in this study cannot be directly correlated with a DC subset that is present in vivo. Nevertheless, this data reinforces the existence of different moDC populations and their relevance. Interestingly, CD1a^−^/CD86^−^/CLEC12A^+^ moDCs supported viral gene expression to a larger extent than the other two subsets. Moreover, CD1a^−^/CD86^−^/CLEC12A^+^ moDCs appeared to be the least suited subset for antigen presentation and showed high expression of the tolerogenic chemokine CCL18^56,57^. Thus, it is tempting to speculate that increased infection of tolerogenic DCs is a mechanism deployed by HCMV to induce regulatory T cells, which would facilitate lytic and latent infection^58,59^. In conclusion, our results show complex interactions between HCMV and moDCs, in which moDC intrinsic gene expression profiles, and in particular expression of the key factor STING, viral load, and the modulation of IFNs and ISGs by the virus are critical determinants for the onset, speed and progression of productive HCMV infection.

## Methods

Research performed in this study complies with all relevant ethical regulations (ethics committee of the Hannover Medical School, ethical approval no. 8315_BO_K_2019) and received informed consent from all participants.

### Primary cell isolation and in vitro differentiation of monocyte-derived dendritic cells

Blood samples of healthy donors were obtained from the Blutspendedienst NSTOB (Niedersachsen-Sachsen-Anhalt-Thüringen-Oldenburg-Bremen gGmbH, Institut Springe) and the Institute of Transfusion Medicine and Transplant Engineering, Hannover Medical School, Germany (this work was approved by the ethics committee of the Hannover Medical School, ethical approval no. 8315_BO_K_2019). CD14^+^ monocytes were isolated from PBMCs by MACS sorting. For differentiation of monocyte-derived dendritic cells (moDCs), and GM-CSF, as well as M-CSF macrophages, 5 ×10^5^ cells/ml of the monocytes were cultivated for 5 days in serum-free CellGenix® GMP DC medium (CellGenix) enriched with 1000 U/ml GM-CSF (granulocyte macrophage-colony stimulating factor, Miltenyi) and 1000 U/ml IL-4 (interleukin 4, Miltenyi) or 80 U/ml GM-CSF, or 100 ng/ml M-CSF (macrophage-colony stimulating factor, Miltenyi Biotec), respectively.

### Virus

In this study two different HCMV reporter viruses were used. In the reporter HCMV strain TB40/E-UL122/123-mNeonGreen (HCMV-NG), the mNeonGreen cassette is linked via a P2A peptide sequence with the UL122/123 coding region. As the P2A peptide induces ribosomal skipping, the major immediate early promoter (MIEP) drives transcription of one mRNA encoding both the mNeonGreen and UL122/123, and the translation of this mRNA into mNeonGreen is terminated at the end of the P2A peptide and re-initiated for UL122/123. As a consequence, mNeonGreen and UL122/123 proteins are formed at an equimolar ratio, and the proteins are folded independently^20^. In the triple-reporter HCMV strain TB40/E-UL122/123-mNeonGreen/UL112/113-mTagBFP2/UL48A-mCherry (HCMV^3F^), the mNeonGreen cassette was inserted as described above, whereas the mTagBFP2 cassette linked to the P2A peptide sequence was fused with a nuclear localization sequence (NLS) and inserted before the first exon of the UL112/UL113 coding region. For the late reporter, the mCherry cassette followed by the T2A peptide sequence (which is another 2A peptide) was inserted between the start codon and the first exon of the UL48A coding region^24^. Correspondingly, expression of the reporter genes included in HCMV-NG and HCMV^3F^ faithfully reflect viral gene expression, despite the reporters are no fusion proteins with viral components.

Both reporter viruses were propagated in lung fibroblasts (MRC-5, ATCC® CCL-171™) and were concentrated from cells and supernatants by centrifugation at 25,000 x *g*, 10 °C for 3 h and additionally purified using 20% sorbitol gradient centrifugation at 53,000 x *g*, 10 °C for 1 h 15 min. Infectious virus yields were determined on MRC-5 cells as described previously^60^.

### Virus titers

Viral titers were determined on MRC‐5 cells by an indirect immunoperoxidase staining procedure^61^. In brief, MRC‐5 cells were infected with 10‐fold serial dilutions of HCMV, centrifuged at 300 x *g* for 30 min and incubated for 3 days. MRC-5 were fixed with methanol for a minimum of 1 h at −20 °C. The cells were incubated for 30 minutes with the primary antibody directed against cytomegalovirus immediate early and early nuclear proteins (#M0854, 1:100, Dako) and then for another 30 minutes with the secondary goat anti‐mouse‐HRP antibody (#5450-0011, 1:500, KPL). Afterwards the cells were incubated with the substrate AEC (#925804 and #925903, 1:50, Biolegend) for 20 minutes. Infected cells were microscopically counted and viral titers were calculated. These titers were used to calculate the MOI for the infection of moDCs and cell lines.

### Infection procedures

For infection of moDCs, the cells were either mock-treated or HCMV-NG exposed at MOI 6. In some experiments, HCMV-NG was UV-inactivated using 300 mJ/cm^2^ prior to moDCs exposure. Viral entry was enhanced by centrifugation at 300 x *g* for 30 min. scRNA-seq was performed at 8 hpe and flow cytometry analysis was performed at 8 hpe and 24 hpe.

### Flow cytometry analysis and cell sorting

moDCs were harvested and stained with Zombie Aqua™ Fixable Viability Kit (BioLegend) in cold PBS. Surface marker immunolabeling was performed in cell staining buffer (PBS, BSA, EDTA) with the following antibodies: CD1a, CD85d, CD86, CD88, CD115 and CLEC12A (1:20, BioLegend). Immunolabeling of the cells was performed for 20 min at 4 °C. Intracellular CCL17 (1:10, R&D Systems), CCL18 (1:10, Miltenyi Biotec), and CCL22 (1:10, BD biosciences) immunolabeling was performed according to the intracellular labeling protocol from BD Bioscience. Then the samples were acquired on a SP6800 or ID7000 (Sony) and the data were analyzed using FlowJo software (Tree Star). RFI was calculated as the ratio of the mean fluorescence intensity (MFI) of the antibody labeling and the isotype labeling.

For cell sorting, moDCs were harvested on day 5 of differentiation, and stained with Viobility™ Fixable Dye (Miltenyi) or immunolabeled with anti-CD1a and anti-CD86 antibodies (1:20, BioLegend). Sorting of CD1a^+^, CD86^+^, and CD1a^−^/CD86^−^ cell subsets was performed using the MACSQuant® Tyto® sorter (Miltenyi). Afterwards, cells were plated in fresh CellGenix® GMP DC medium (CellGenix) for 24 h and subsequently infected with HCMV-NG for 24 h. Sorting of NG^+^ and/or NG^−^ moDCs was performed using the FACSAria Fusion sorter (BD). Afterward, cells were plated in fresh CellGenix® GMP DC medium (CellGenix) for 24 h or plated on top of MRC-5 fibroblast monolayers for 4 days.

### ELISA analysis

Cell-free supernatants were analyzed using the Human IFN-alpha Platinum ELISA (Thermofisher), human IFN Beta, and Lambda ELISA kit (PBL) according to the manufacturer’s instructions.

### In vitro treatment with cytokines and STING agonists

Treatment with 10 µM ADU-S100 (purchased from MedChem express), 1 ng/ml TNF (Miltenyi), 100 U/ml IFN-α2b (IntronA, MSD), 1 ng/ml IFN-β and 10 ng/ml IFN-λ1 (Peprotech) was performed 1 day prior to HCMV-NG exposure (-1 dpe) or at time of virus exposure (0 dpe) at 37 °C. Subsequently, samples were harvested 1 day post HCMV-NG exposure as described above to determine NG^+^ cell percentages by flow cytometry.

### Subcellular fractionation, RNA extraction, and qPCR analysis

The subcellular protein fractionation kit (#78840; Thermo Fisher Scientific) was used for fractionating moDCs into three fractions (cytoplasmic extract, membrane extract, and soluble nuclear extract), of which the nuclear fraction was used for analysis. gDNA was extracted from the nuclear fraction using QlAamp DNA 161 Blood Mini Kit (#51106; Qiagen) and qPCR analysis was performed using the following primers: hMDM2 forward, 5ʹ-GGTTGACTCAGCTTTTCCTCTTG -3ʹ and reverse, 5ʹ GGAAAATGCATGGTTTAAATAGCC-3ʹ; HCMV *UL44* promoter forward, 5ʹ AACCTGAGCGTGTTTGTG-3ʹ and reverse 5ʹ-CCCGACTAAGAGGCACAGTA-3ʹ^28^.

### CITE-seq labeling, single-cell library preparation, and sequencing

moDCs from two healthy, HCMV seronegative, male donors were prepared and infected as described above. Afterwards, mock treated and HCMV-NG exposed cells were CITEseq labeled with TotalSeq^TM^-B antibodies anti-human CD45 (anti-CD45-ADT, mock treated cells) or anti-human HLA-DR antibody (anti-HLA-DR-ADT/ HCMV-NG exposed cells) (#304066 and # 307661, respectively; BioLegend) according to the manufacturer’s protocol for TotalSeq^TM^-B antibodies with 10×3’ Reagent Kit v3.0 Feature Barcoding Technology.

In brief, 1 × 10^6^ mock treated and HCMV-NG exposed cells from each of the four groups (1×10^6^ cells/ml) were resuspended separately in 50 µl Cell Staining Buffer (BioLegend). Subsequently, 5 µl of Human TruStain FcX™ Fc Blocking reagent (BioLegend) was added and incubated for 10 min at 4 °C. After the incubation, 1 µg anti-human CD45 or anti-human HLA-DR TotalSeq-B antibodies were added to the cell suspension and incubated for 30 min at 4 °C. The cells were washed in 1.5 ml staining buffer and centrifuged at 400 x *g* and 4 °C for 5 min. Subsequently, the cells were resuspended in an appropriate volume of 1x DPBS (Gibco), passed through a 40 µm mesh (FlowmiTM Cell Strainer, Merck), and counted, using a Neubauer counting chamber (Marienfeld). 1/3 of mock-treated cells was pooled with 2/3 of HCMV-NG exposed cells. Importantly, mock-treated cells that were derived from the same sample were combined with HCMV-NG V1 and V2 exposed cells to provide an internal control for batch effects.

Labeled cell suspensions were loaded in the Chromium^TM^ Controller (10x genomics). Single Cell 3’ reagent kit v3.1 was used for reverse transcription, cDNA amplification, and library construction following the detailed protocol provided by 10x Genomics. CITE-seq libraries were prepared according to the Feature barcoding protocol for 10x Single Cell 3’ Reagent Kit v3.

SimpliAmp Thermal Cycler was used for amplification and incubation steps (Applied Biosystems). Libraries were quantified by Qubit^TM^ 3.0 Fluometer (ThermoFisher) and quality was checked using 2100 Bioanalyzer with High Sensitivity DNA kit (Agilent). Sequencing was performed in paired-end mode with an S2 2 × 50 cycles kit using a NovaSeq 6000 sequencer (Illumina).

### Sequencing of virus preparations

To analyze the virion-associated RNA within the employed virus preparations, RNA was extracted from 1.3 × 10^8^ plaque-forming units per ml of virus stock with the Quick-RNA Viral kit (Zymo) by using 10 μl from 2 independent viral preparations (V1 and V2). RNA was eluted in 6 μl nuclease-free water and 3 μl was used for the Smart-seq v4 low input reaction (Takara) with one-quarter of the recommended reagent volumes. ERCC spike-in control was added to a dilution of 1:20 million. Libraries were prepared using Nextera XT (Illumina) using a quarter of the recommended reagent volumes, pooled and sequenced in paired-end mode on the NextSeq 500 sequencer (IIIumina) using the Mid Output 2 × 75 cycle kit.

### Single-cell read mapping and counting

We used the 10x Genomics CellRanger software (version 3.0.2) to map the “Gene expression” libraries against a combined index of the human (HG38, Ensembl v90 annotations; filtered to include only “protein_coding”, “lincRNA”, “antisense”, “IG” and “TR” genes, as recommended in the CellRanger documentation) and HCMV (GenBank accession: EF999921), which was adapted to include the NeonGreen cassette in the viral genome. The TotalSeq-B antibody libraries were mapped against the CellRanger internal index. Using an in-house genome browser, we then went through the viral genome and manually annotated significant clusters of reads at polyadenylation sites (Supplementary Fig. 2a). We created a new combined index of human (see above) and these new annotations of read clusters as a replacement of the annotated viral open reading frames and ran CellRanger again to obtain the final expression matrices. Overall, this resulted in 26,395 cells after the default filtering from CellRanger.

### Processing of Smart-seq data from virus preparations

SMART-seq data from the two virus preparations were mapped against the combined index generated by CellRanger using STAR (version 2.5.3a)^62^ with standard parameters. Since these data contained reads not only from the 3’ ends of mRNAs, we adapted the cluster annotations for read counting as follows. We identified the closest cluster upstream of each cluster to define the upstream distance. If this was greater than 1,000 nt, we set it to 1,000. Then, each cluster was extended in the upstream direction by its upstream distance. We manually compared these annotations with the observed read coverages in a genome browser to confirm these manual annotations.

### Demultiplexing, quality control and preprocessing of 10x scRNA-seq data

Centered log-ratio normalization was applied to the TotalSeq counts and strict thresholds for demultiplexing were identified in a scatterplot of the CD45-ADT value vs. the HLA-DR-ADT value. We decided to call all cells with HLA-DR-ADT value > 0.35 as HCMV-NG exposed, and all cells with CD45-ADT value > 0.9 as mock-treated. Double negative cells (*n* = 1,151) and doublets (*n* = 869) were removed. We further removed all cells with less than 10,000 or more than 40,000 detected UMIs and with more than 17% mitochondrial RNA, which left us with 13,566 HCMV-NG exposed and 5270 mock-treated cells.

We then used the SCTransform pipeline to normalize the remaining cells^63^ and performed principal component analysis. Based on elbow plot analysis we used the first 45 principal components to compute the shared nearest neighbor graph and performed Louvain clustering (resolution 0.8)^64^. The UMAP algorithm^65^ was run on the first 45 principal components with standard parameters.

Two clusters were identified to be composed of doublets and were removed. Unsupervised clustering generated clusters that in most cases donor-specific but in some cases, they contained cells from both virus preparations. Thus, the clustering was not due to batch effects, but it highlighted biological differences between the two donors. Only clusters 3 in CD45-ADT^+^ cells and clusters 8 and 9 in HLA-DR-ADT^+^ cells (see Fig. 1c, d) were clusters composed of cells from both donors. To be able to show the differences and commonalities of the two donors, we split these clusters according to donor origin. For all other clusters, one donor was dominant (>95% of all cells), and cells from the other donor were removed.

### Data integration by canonical correlation analysis

We used canonical correlation analysis (554) as described^66^ and implemented in Seurat (IntegrateData function) to identify relationships between single cells from one defined subset of cells in our data with cells from another subset based on the correlation structures of automatically selected integration genes. In each case, viral genes were not considered as integration genes, i.e., they were removed from the integration features selected by Seurat prior to integration.

To integrate the mock-treated cells (Fig. 1c, we first removed all “B” and “P” clusters and split all remaining cells according to donor information. Thus, IntegrateData was run to integrate two subsets of cells, the mock-treated cells from donor 1 and the mock-treated cells from donor 2. Accordingly, to integrate bystander and productively infected cells (Fig. 1d, e, respectively), we removed the mock-treated cells and again split all remaining cells according to donor origin. Thus, IntegrateData was run to integrate again two subsets of cells, namely all B and P cells from donor 1, and all B and P cells from donor 2.

To compute the alluvial charts (Fig. 6c, d), analyses were performed separately for donor 1 and donor 2. For each donor, we split all cells into the M and B + P subsets and then used IntergrateData to integrate these two subsets. Then, to compute the composition of all B and P cell clusters according to their corresponding cluster in the mock-treated cells (which is shown in the alluvial chart), we computed the k nearest neighbor graph (with k set to 5% of all cells used) for the integrated data set. Each bystander or productively infected cell was then assigned to a mock-treated cluster based on a majority vote among the nearest neighbors.

### RNA velocity and ISG unspliced vs. spliced analysis

We first ran the run10x command from the Velocyto package^25^ to extract count matrices for spliced and unspliced reads from the bam files generated by CellRanger. RNA velocities were then computed using the Velocyto R package with kCells set to 15, and the velocity plot was generated by calling the show.velocity.on.embedding.cor function on the UMAP computed by CellRanger.

To compute the unspliced over spliced ratio for ISGs, we first computed the 20 nearest neighbors for each cell and then computed the convolution for both spliced and un-spliced count matrices by matrix multiplication of the adjacency matrix of the nearest neighbor graph. The total spliced or un-spliced value for each cell was then computed as the total sum of all HALLMARK_INTERFERON_ALPHA_RESPONSE genes from MSigDB.

### Pathway enrichment

For pathway analysis, we first computed marker genes for each donor separately using the fast Wilcoxon test (wilcoxauc function from the presto package). We then ran the gsva function from the GSVA R package^67^ using the average expression values per cluster on a predefined set of pathways. All selected pathways were chosen from MSigDB and can be found in Fig. 6f, Supplementary Fig. 6f and Supplementary Data File 4. To compute statistical significance, we randomly permuted each column in the genes cluster matrix and rerun GSVA. This was repeated 100 times. We then computed the mean and standard deviation for the GSVA scores of each pathway and each cluster of the 100 permutations and used this to compute the z score of the corresponding GSVA score from the non-permuted matrix. The z score was converted into a two-sided P value using the standard normal distribution, and false discovery rates were obtained by multiple testing corrections using the Benjamini-Hochberg procedure.

### Correlation and fold change analysis

To compute fold changes between HCMV-NG and mock-treated cells we extracted the averages per cluster from the SCTransform-normalized data. The fold changes of the HCMV-exposed vs. mock-treated cells were then computed by subtracting the logarithmized (base 2) mean of all M clusters of these averages from the logarithmized mean of all other clusters. The fold changes of productively infected cells over bystander cells were extracted from the wilcoxauc output used to compute marker genes (presto package). Spearman correlations with the total percentage of viral gene expression for each gene were computed using the base cor R function. The gene set enrichment test was computed using a two-sided Wilcoxon test comparing the fold changes or correlation coefficients from genes that belong to a particular gene set against genes not belonging to the gene set. Only the highly variable genes defined by SCTransform and all gene sets from the Hallmark category of MSigDB^68^ were considered (Supplementary Data File 2).

The Spearman correlations of viral or cellular genes with *IFNB1* or *IFNL1* expression (Supplementary Data File 3 and 4) or the ISG inhibition value (unspliced over spliced RNA) were computed using base cor R function using the cells from the productively infected cluster only.

### Statistics and reproducibility

Statistical tests were performed using GraphPad Prism 9 Vers. 9.3.1 (GraphPad Software Inc) as indicated in the respective figure legends. Reproducibility of experiments was determined in independent experiments as indicated in the figure legends. No statistical method was used to predetermine sample size. No data were excluded from the analyses. The experiments were not randomized. The Investigators were not blinded to allocation during experiments and outcome assessment. Detailed descriptions of the analyses and statistical methods used for analysis of the scRNA-seq data are found in the respective methods sections above.

### Reporting summary

Further information on research design is available in the Nature Portfolio Reporting Summary linked to this article.

### Supplementary information

Supplementary Information

Description of Additional Supplementary Files

Supplementary Data 1

Supplementary Data 2

Supplementary Data 3

Supplementary Data 4

Supplementary Data 5

Reporting Summary

### Source data

Source data

## Data Availability

Data can be accessed via Zenodo 10.5281/zenodo.10404879 (https://zenodo.org/records/10404879). The raw sequencing data are protected due to the data protection act (DPA) and can only be accessed upon request. Data can be browsed via the web interface http://einstein.virologie.uni-wuerzburg.de:3839/45559dc12750521deffaff3b105e9615/. Source data are provided with this paper.

## Code availability

Codes can be accessed via Zenodo 10.5281/zenodo.10404879 (https://zenodo.org/records/10404879).
