# Supplementary material for: Human cytomegalovirus exploits STING signaling and counteracts IFN/ISG induction to facilitate infection of dendritic cells
Source: Nat Commun. 2024 Feb 26;15:1745. doi: 10.1038/s41467-024-45614-3 (PMC10897438; doi:10.1038/s41467-024-45614-3)
Supplement: Supplementary file 1 — Supplementary Information [file 41467_2024_45614_MOESM1_ESM.pdf]

Supplementary information

Human cytomegalovirus exploits STING signaling and counteracts IFN/ISG induction to facilitate infection of dendritic cells

This file includes Supplementary Fig. 1 to 7 and figure legends.

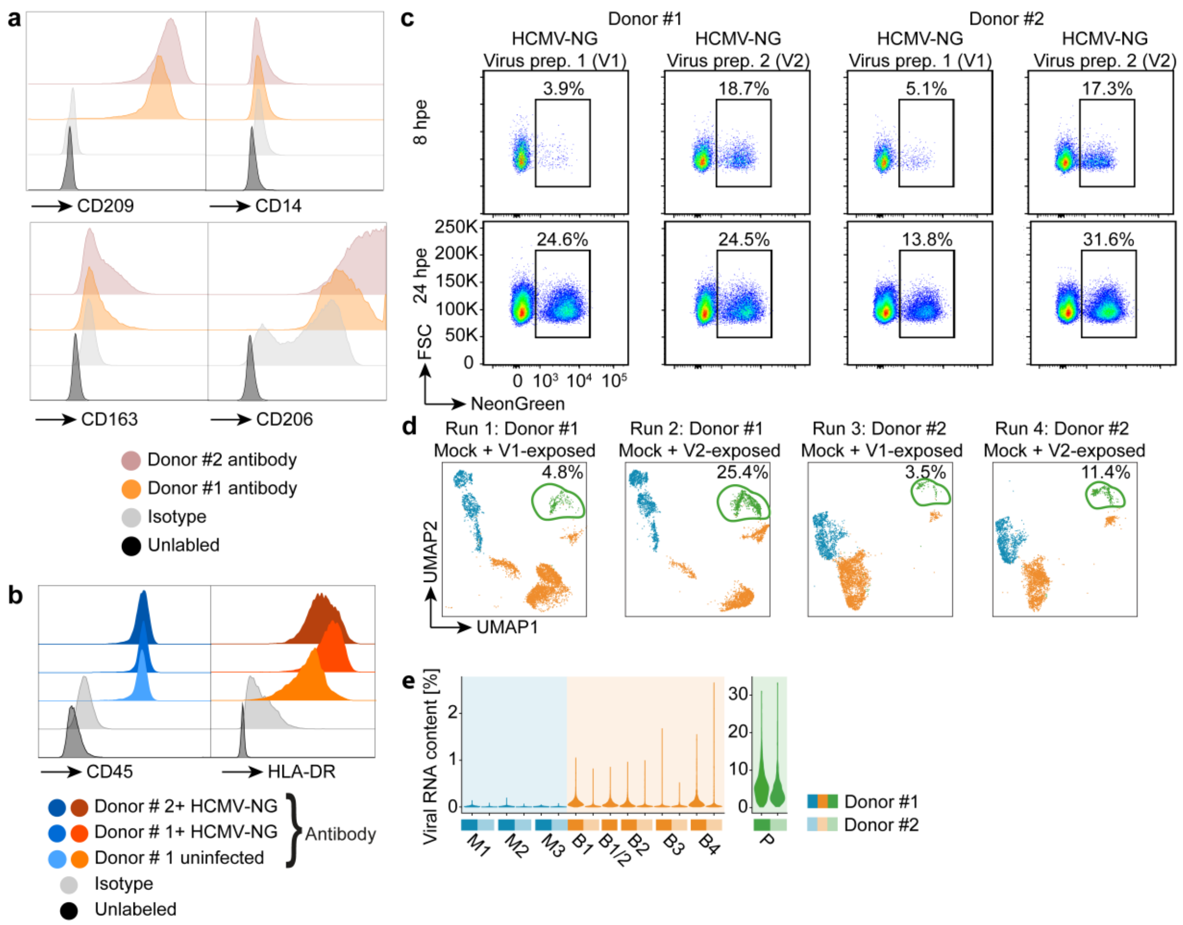

Supplementary Fig. 1: Independent scRNA-seq runs confirm a high degree of similarity between the two tested donors.

moDCs were differentiated from blood monocytes of two donors (donor #1 and donor #2) and mock treated or HCMV-NG exposed using two independent HCMV-NG preparations (V1 and V2). **a** Expression of monocyte and moDCs canonical markers CD209, CD14, CD163, and CD206 and **b** expression of CD45 and HLA-DR measured by flow cytometry on cells from the donors used in the scRNA-seq experiments. **c** Mock treated and HCMV-NG exposed cells were pooled prior to sequencing. Thus, a total of 4 runs were sequenced. In parallel, moDCs from the same donors were analyzed for percentages of HCMV-NG^{+} cells at 8 hpe (upper panel) and 24 hpe (lower panel) using flow cytometry. Dot plots of moDCs from donor #2 infected with HCMV-NG V2 are the same as shown in Fig. 1a. **d** UMAP embeddings of the 4 runs of sequencing at 8 hpe. The strong correspondence between mock treated cells (shown in blue) of run 1 and 2 or run 3 and 4 demonstrate that batch effects between runs were minimal. **e** Distribution of the percentages of viral RNA levels of each cluster is shown as a violin plot.

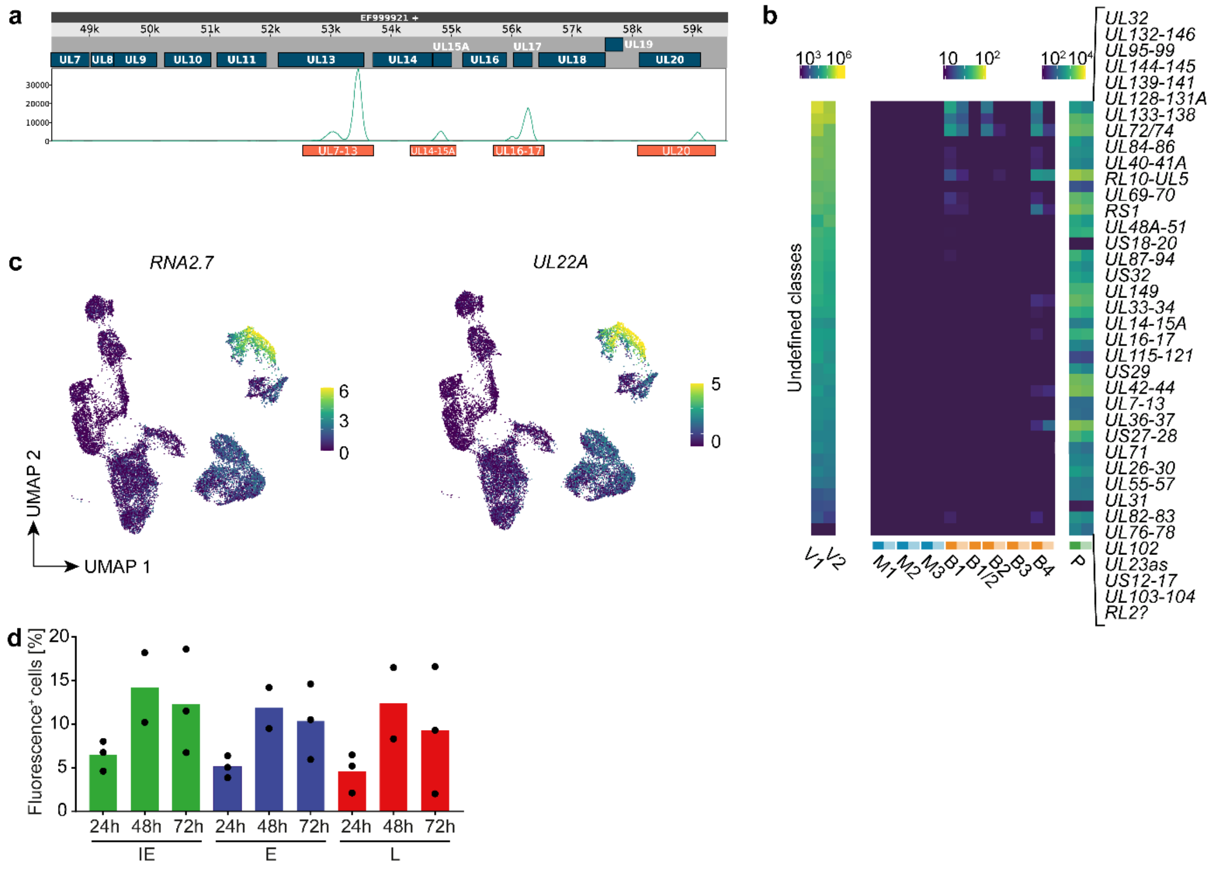

Supplementary Fig. 2: Manual assignment of HCMV genes to individual polyadenylation sites.

a Genome browser screenshot for the *UL7-20* locus of the HCMV genome. Blue boxes represent open reading frames, the green line is the coverage of pooled scRNA-seq reads, and orange boxes indicate the manually curated cluster annotations. **b** Mapping and quantification of HCMV RNAs with undefined kinetics in viral preparations (V1, V2) used for the infection experiments as well as mock treated (M), bystander (B), and productively infected (P) moDCs. **c** UMAPs showing the abundance of the virion-associated RNAs *RNA2.7* and *UL22A*, in the sequencing data. **d** Quantification of fluorescence in moDCs exposed to HCMV^{3F} at MOI 3. Percentages of NG (green bars), BFP (blue bars), and mCherry (red bars) fluorescence of cells were analyzed by flow cytometry at 24, 48, and 72 hpe from a total of three different donors from one experiment.

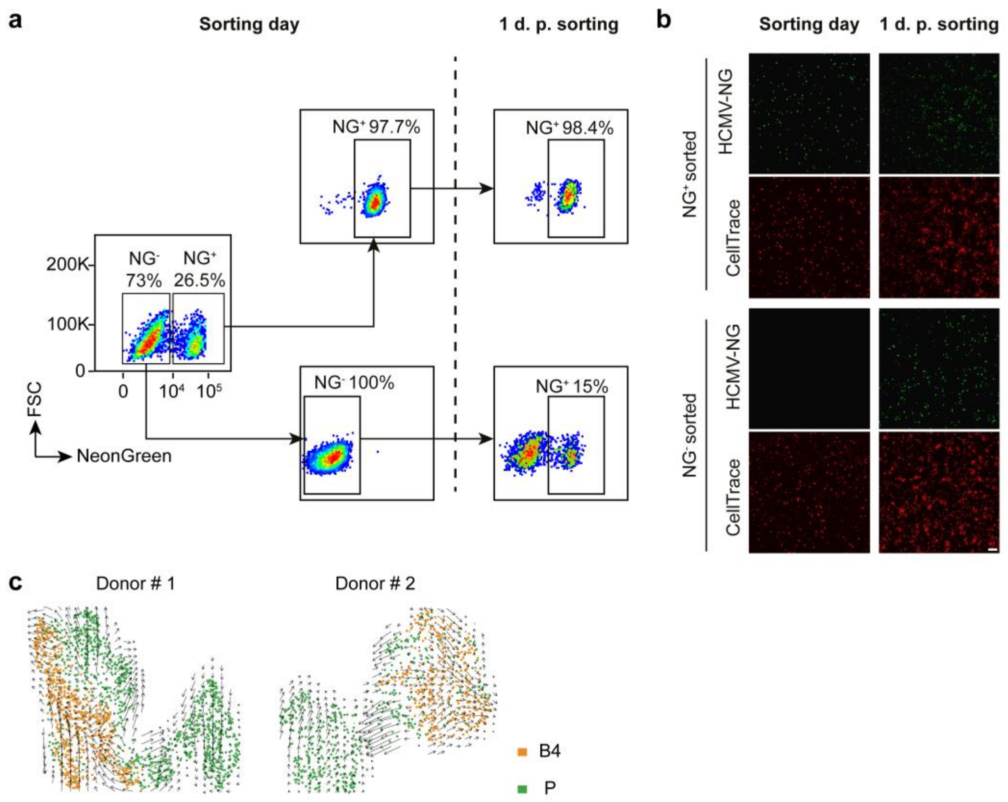

Supplementary Fig. 3: Upon HCMV-NG exposure, most moDCs are infected and after sorting of NG$^-$ cells IE gene expression can be re-initated.

a, b Flow cytometric (**a**) and fluorescence microscopic (b) analysis of moDCs exposed to HCMV-NG and sorted for NG$^-$ and NG$^+$ fluorescence. NG$^+$ cells are shown in green and live cells are stained by CellTrace and shown in red (scale bar 100 µm). Data from one out of two donors is shown. **c** RNA-velocity analysis of bystander cells in B4 (orange) and productively infected cells in P (green). Arrows indicate future trajectories of the cells.

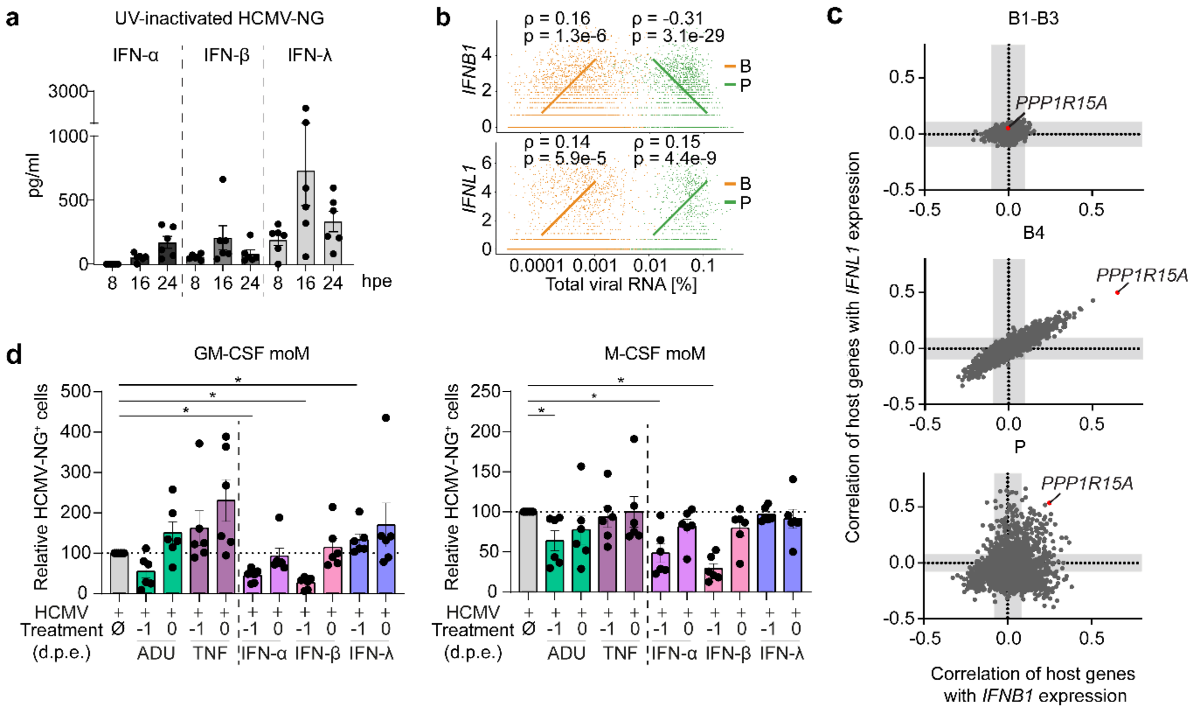

Supplementary Fig. 4: IFN-β and IFN-λ are expressed prior to IFN-α in HCMV-NG exposed moDCs.

a moDCs were exposed to UV-inactivated HCMV-NG and supernatants were harvested completely and replenished with fresh medium 8, 16 and 24 hpe to determine the IFN-α (black bars), IFN-β (dark grey bars) or IFN-λ (light grey bars) content by ELISA methods. **b** Expression (SCTransform normalized values) of *IFNB1* and *IFNL1* (y-axis) compared to total viral RNA abundance (x-axis, in percentage relative to total feature counts per cell) in bystander (orange) and productively infected (green) cells. Values indicate Spearman's correlation coefficients as determined in Fig. 4b and lines visualize a positive or negative correlation. **c** Spearman´s correlation coefficients of host genes with *IFNB1* (x-axis) and *IFNL1* (y-axis) expression for clusters B1-3 (1st graph), B4 (2nd graph), and P (3rd graph) for each individual. White areas indicate statistically significant regions (p<0.01, approximate t-test, Benjamini-Hochberg multiple testing correction). **d** GM-CSF (left graph) and M-CSF (right graph) monocyte derived-macrophages (moM) were treated at 1 day before HCMV-NG exposure (-24 hpe) and at the time of HCMV-NG exposure (0 hpe) with IFN-α2b (lavender bars), IFN-β (pink bars) or IFN-λ1 (blue bars) or ADU-S100 (ADU) (green bars), and tumor necrosis factor (TNF) (purple bars), and percentages of NG+ cells were quantified by flow cytometry. Mean ± SEM of 6 different donors from 3 independent experiments. Each dot represents a single donor. *: p≤0.0313 using two-tailed paired Wilcoxon signed-rank test.

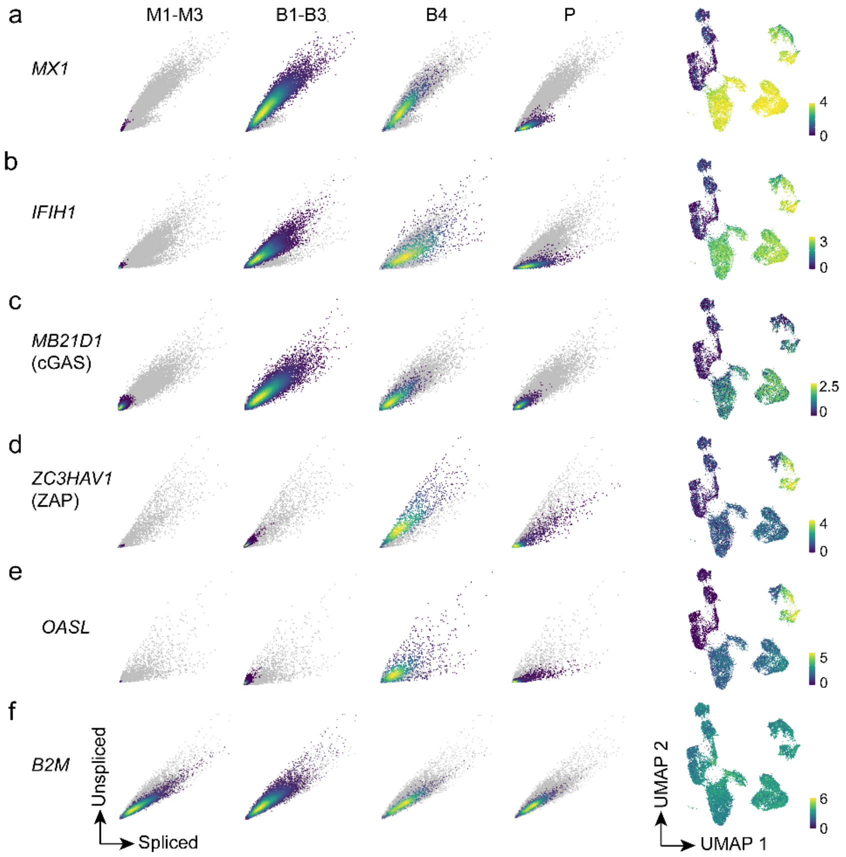

Supplementary Fig. 5: ISG transcription is discontinued in productively infected cells.

Phase portraits showing expression of unspliced (y-axis) and spliced (x-axis) reads for **a** *MX1*, **b** *IFIH1*, **c** *MB21D1* (cGAS), **d** *ZC3HAV1* (ZAP), **e** *OASL* and **f** *B2M* RNAs per cell. Cell densities from clusters M1-3 (1st graph), B1-3 (2nd graph), B4 (3rd graph), and P (4th graph) are highlighted in color. Additionally, UMAPs of spliced RNAs of each gene are shown.

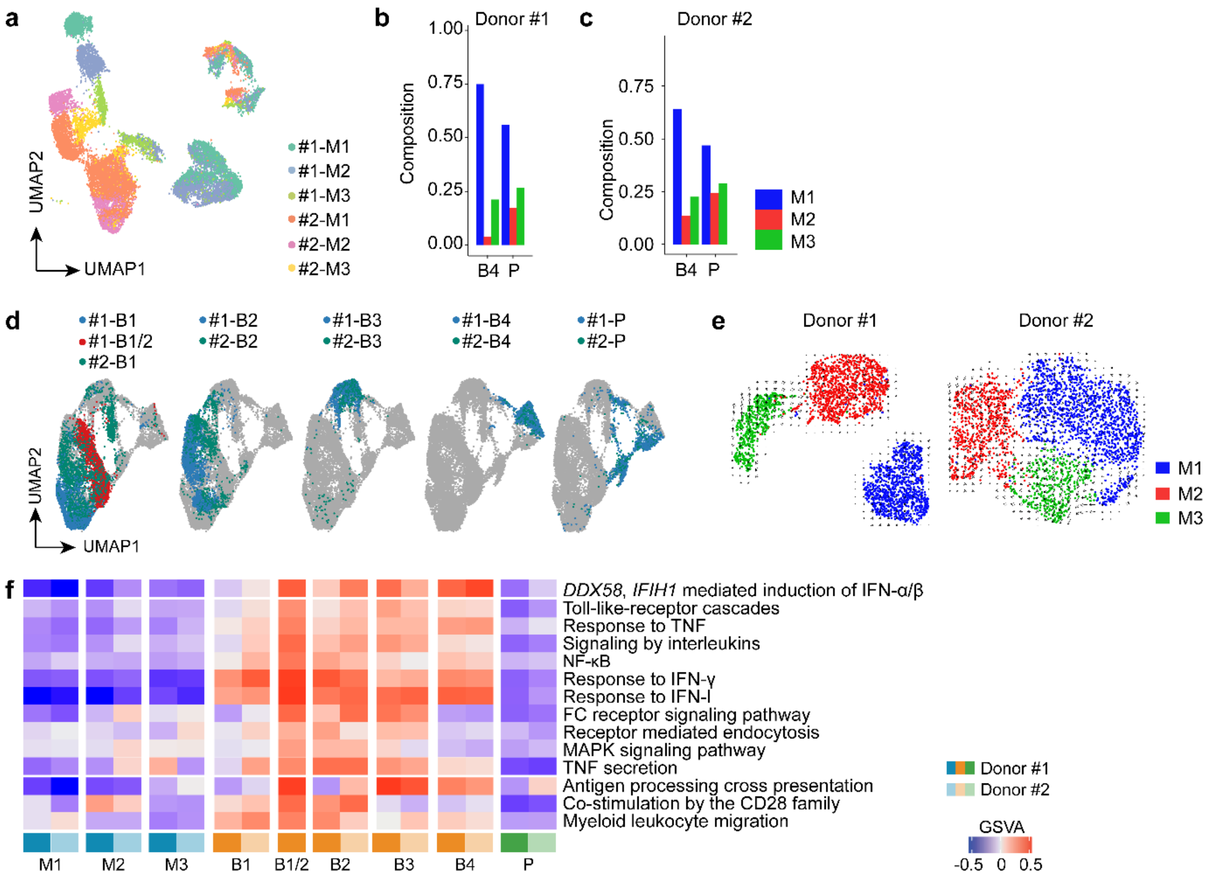

Supplementary Fig. 6 moDCs are composed of three different clusters with distinct gene expression profiles that are detected under conditions of homeostasis and after HCMV-NG exposure.

a UMAP showing correspondence between mock treated and the respective bystander and productively infected moDCs. Colors indicate the actual cluster assignment for mock treated cells and the cluster of origin for cells in B and P as inferred from canonical correlation analysis. **b, c** Composition of cells in B4 and P of donor #1 (**b**) and donor #2 (**c**) originating from clusters M1-3 of mock treated cells, as determined by canonical correlation analysis, normalized for the different cluster sizes of M1-M3. **d** Integration of HCMV-NG exposed cells from both donors by canonical correlation analysis. B1, B2, B3, B4, and P clusters of each donor are highlighted in separate UMAPs. **e** UMAP embedding of M1, M2 and M3 of donor #1 and #2 with their future trajectories (arrows) estimated by RNA velocity analysis. **f** Pathway analysis of manually selected DC characteristics for the different clusters using gene set variation analysis (GSVA). Positive GSVA scores indicate an enrichment of strongly expressed genes in the respective pathways. Pathways shown here and in Fig. 6h are those which were included in the analysis.

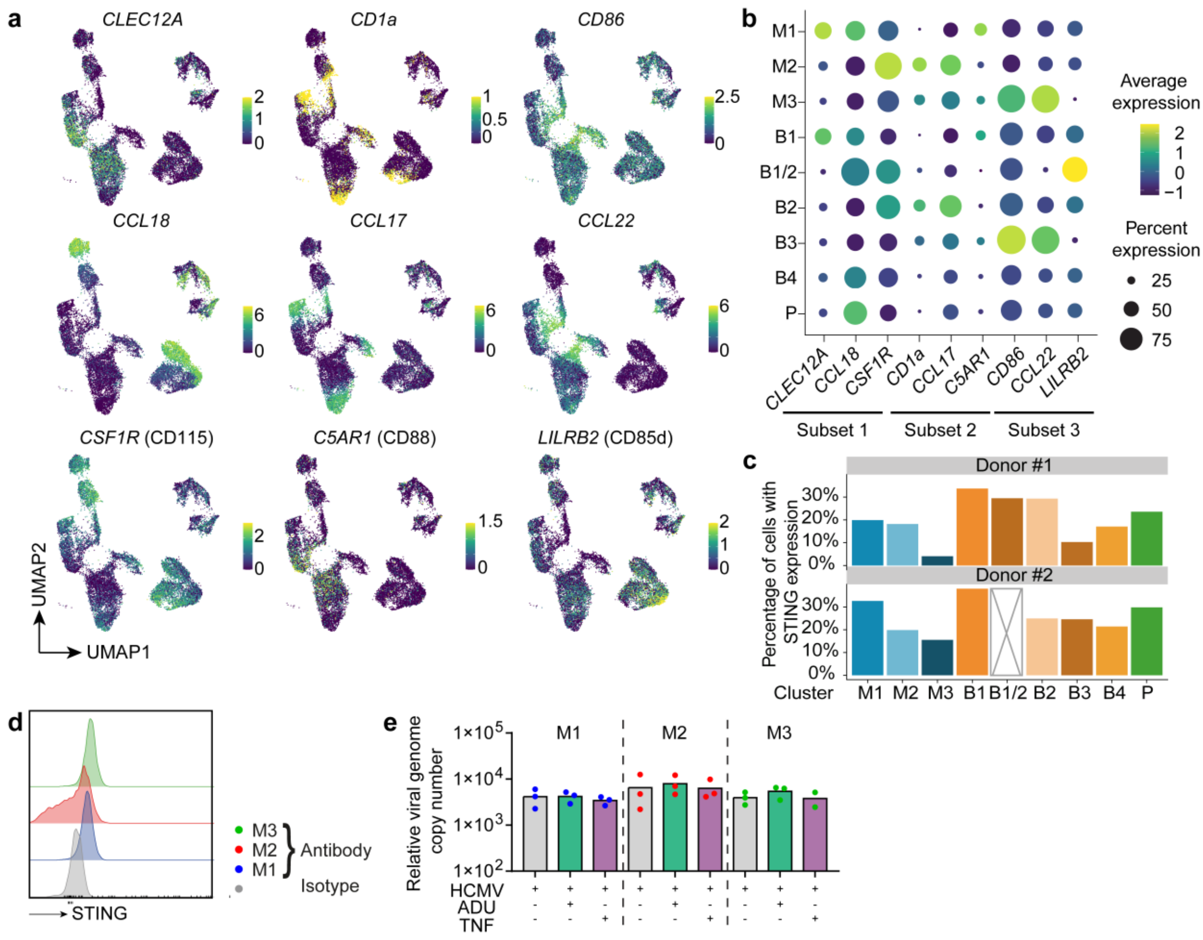

Supplementary Fig. 7: moDC subsets express discriminating markers.

a UMAPs showing the expression of discriminative markers in the different moDC subsets. Expression of these markers was determined on the protein level in Fig. 7a. **b** Dotplot of the expression of discriminating markers across the transcriptionally defined moDC subsets. Cumulated data of both donors. **c** Bar plots showing the distribution of the percentages of cells expressing STING RNA in each mock treated (blue bars), bystander (orange bars), and productively infected (green bars) clusters for the two donors. The empty bar represents the cluster that is absent in donor #2. **d** Flow cytometry analysis of STING protein expression in the three moDC subsets during homeostasis from one representative donor. **e** M1 (blue dots), M2 (red dots), and M3 (green dots) moDC subsets were treated with STING agonists (ADU) (green bars) or with a NF-κB agonist (TNF) (purple bars) at the time of HCMV exposure. After 4 h incubation, the genomic DNA was extracted from the soluble nuclear fraction and the relative abundance of HCMV genomes in relation to the housekeeping gene GAPDH was analyzed by qPCR.
