# Supplementary material for: Human cytomegalovirus exploits STING signaling and counteracts IFN/ISG induction to facilitate infection of dendritic cells
Source: Nat Commun. 2024 Feb 26;15:1745. doi: 10.1038/s41467-024-45614-3 (PMC10897438; doi:10.1038/s41467-024-45614-3)
Supplement: Supplementary file 2 — Description of Additional Supplementary Files [file 41467_2024_45614_MOESM2_ESM.pdf]

Description of Additional Supplementary Files

Supplementary Data 1: List of marker genes in each cluster shown in Fig. 1f, g where the two-sided Wilcoxon rank sum test was applied. Padj is corrected for multiple testing using the approach by Benjamini and Hochberg.

Supplementary Data 2: Single-cell summary statistics for highly variable genes in Fig. 3b where the two-sided approximate t-test, Benjamini-Hochberg multiple testing correction was applied.

Supplementary Data 3: Spearman's ρ of viral genes vs. IFN-β and IFN-λ gene expression (together with multiple testing corrected p values estimated using the two-sided asymptotic t-test) for bystanders (B1-3 and B4) or productively infected cells (P).

Supplementary Data 4: Spearman's ρ of host genes vs. IFN-β and IFN-λ gene expression (together with multiple testing corrected p values estimated using the two-sided asymptotic t-test) for bystanders (B1-3 and B4) or productively infected cells (P).

Supplementary Data 5: List of pathways used in Fig. 6f and Supplementary Fig. 6f and GSVA, and FDR values used in Fig. 6f.
